# Plant‐protein supplementation improves thermoregulatory responses and ameliorates markers of intestinal damage during exercise in the heat

**DOI:** 10.1113/EP093504

**Published:** 2026-03-31

**Authors:** Robyn Aitkenhead, Mark Waldron, Gillian E. Conway, Katy Horner, Shane M. Heffernan

**Affiliations:** ^1^ A‐STEM Centre, Faculty of Science and Engineering Swansea University Swansea UK; ^2^ Welsh Institute of Performance Science Swansea University Swansea UK; ^3^ In Vitro Toxicology Group, Institute of Life Science Swansea University Medical School Swansea UK; ^4^ School of Public Health Physiotherapy and Sport Science and UCD Institute of Food and Health University College Dublin Dublin Ireland

**Keywords:** amino acid, exercise‐induced gastrointestinal syndrome, fava bean, plant‐based food, thermoregulation

## Abstract

Exercise in the heat often causes gastrointestinal (GI) disturbances, which can impair performance. Single amino acid supplementation can attenuate gut damage and enhance exercise tolerance; however, the effectiveness of innate amino acid blends from plant‐based proteins remains underexplored. In this study, we investigated the effects of a novel fava bean‐derived plant protein (ATURA) on thermoregulation, endurance performance and GI disturbances during exercise. Twelve healthy, non‐heat‐acclimatized participants completed an exercise trial (10 min walk, 40 min run, graded exercise test) in the heat (35°C; 40% relative humidity), before and after 8 days of ATURA (60 g/day) or placebo, in a double‐blind, randomized, cross‐over design. Whole‐body sweat rate, local sweat rate, pulmonary gas exchange, skin and core temperature and perceptual responses were monitored, with pre‐ and post‐trial blood samples. ATURA increased whole‐body sweat rate (11%; *P =* 0.03) and back local sweat rate (11%; *P <* 0.001) and reduced core temperature (ATURA, 38.7°C ± 0.5°C; placebo, 38.8°C ± 0.5°C; *P =* 0.04) and thermal sensation (*P =* 0.05) in comparison to placebo. There were no differences for skin temperature, thermal comfort or graded exercise test time (*P* > 0.05). ATURA reduced postexercise intestinal fatty‐acid binding protein (22%) compared with placebo (*P =* 0.05), with no difference for soluble CD14 or GI symptoms. Pre‐to‐post HSP70 was higher after ATURA (12%, *P =* 0.05), with no difference for interleukin‐6 (*P >* 0.05). Pre‐exercise fava bean protein reduced postexercise intestinal fatty‐acid binding protein, indicating a potential protective effect on intestinal integrity, and was well tolerated, without increasing GI symptoms. Exploratory outcomes suggest possible thermoregulatory benefits, warranting further investigation.

## Abstract (highlights)

- **What is the central question of this study?**
  Does supplementation with a novel fava bean‐derived plant protein (ATURA) mitigate gastrointestinal disturbances and improve thermoregulation during exercise in the heat?
- **What is the main finding and its importance?**
  ATURA supplementation lowered postexercise markers of gut damage without worsening gastrointestinal symptoms and was associated with higher whole‐body and local sweat rates and thermal sensation. These findings suggest that ATURA protects gut integrity during exercise in the heat and might support thermoregulatory function via an evaporative cooling effect, indicating its potential as an effective pre‐exercise supplement.

## 1 INTRODUCTION

Exercise in hot environments presents significant challenges to gastrointestinal (GI) health, often exacerbating gut damage and symptoms associated with strenuous physical activity (Costa et al., 2022; De Oliveira et al., 2014). The term ‘exercise‐induced gastrointestinal syndrome’ (EIGS) describes a range of GI disturbances that occur in response to exercise stress (Young et al., 2023), particularly when combined with heat exposure. These disturbances are frequently associated with exercise‐related gastrointestinal symptoms (GIS), such as nausea, bloating and abdominal pain, which, in turn, can compromise exercise performance by reducing workload or causing cessation of exercise (Gaskell et al., 2021; Young et al., 2023). The underlying mechanisms of EIGS are complex, involving exertional heat stress that intensifies splanchnic hypoperfusion and hyperthermic injury, contributing to disruptions in intestinal barrier function (Costa et al., 2020). As exercise intensity and duration increase, particularly in hot conditions, there is substantial evidence of increased intestinal injury, permeability, endotoxaemia and systemic inflammation (Henningsen et al., 2024). Specifically, during exercise at high temperatures, a cascade of thermoregulatory disruption begins with heat stress‐induced redistribution of blood flow towards the periphery for heat dissipation (Périard et al., 2021). This simultaneously reduces gut perfusion and impairs the integrity of the intestinal barrier, leading to a compounding physiological stress that progressively challenges exercise tolerance and thermoregulatory capacity as core body temperature continues to rise (Costa et al., 2022; Henningsen et al., 2024).

Emerging dietary strategies have been used to mitigate EIGS, including targeted amino acid (AA) supplementation, which can be consumed in isolated singular form (Pugh et al., 2017; Tataka et al., 2022), in a blend (Costa et al., 2023) or as a whole protein source (Snipe et al., 2017). Whole proteins, in addition to supplying both essential and non‐essential AAs, also naturally provide other nutritional components, such as vitamins, minerals, fat and bioactive properties (Luparelli et al., 2025), which are absent from purified AA supplements and might provide a more comprehensive approach to addressing intestinal injury. Indeed, consumption of whey protein (Snipe et al., 2017) and AA blends (Costa et al., 2023) immediately before and during exertional heat stress has been reported to reduce intestinal fatty‐acid binding protein (i‐FABP), an established biomarker of intestinal injury. Other established biomarkers of EIGS, including systemic inflammatory cytokines and markers of luminal bacterial endotoxin translocation, such as interleukin‐6 (IL‐6), soluble CD14 (sCD14) and lipopolysaccharide, increase in response to exercise stress (Young et al., 2023). However, the potential for protein and AA interventions to mitigate intestinal injury and attenuate downstream systemic responses remains largely unexplored.

Amino acids play essential roles in maintaining intestinal health and function, serving as both essential building blocks and crucial energy sources for the lining of the small intestine (Wang et al., 2009). This is important because the intestinal epithelium acts as a selective barrier against pathogens, in addition to participating in nutrient absorption, metabolism and immune function. Indeed, some AAs appear to support intestinal integrity and might be a useful part of the holistic treatment for GI disorders (Chinevere et al., 2002; Lee et al., 2002; Pugh et al., 2017; Tataka et al., 2022). In humans, acute low‐dose glutamine supplementation (0.25 g/kg of fat‐free mass) reduced markers of intestinal permeability and damage following exercise in hot conditions (Pugh et al., 2017; Tataka et al., 2022). The protective effects of l‐glutamine are thought to be mediated through multiple mechanisms, including the activation of heat shock proteins [HSP70 and heat shock factor‐1 (HSF‐1)] and inhibition of the nuclear factor‐κB pro‐inflammatory pathway (Morrison et al., 2006; Singleton & Wischmeyer, 2006). Additionally, l‐arginine and l‐citrulline have the potential to improve intestinal blood flow and mitigate exercise‐associated hypoperfusion by stimulating the nitric oxide (NO) pathway, which promotes vasodilatation in the intestinal microvasculature and might help to maintain gut perfusion during exertional stress (Costa et al., 2014; Van Wijck et al., 2014). Evidence suggests that AAs such as l‐glutamine, l‐arginine, l‐tryptophan and l‐threonine might each contribute beneficially to gut barrier function by enhancing barrier integrity (Zuhl et al., 2014; Rao & Samak, 2011; Varasteh et al., 2018) and facilitating epithelial repair (Wang et al., 2015; Rao & Samak, 2011). Although these compounds might act in parallel to support gut health, the potential synergistic effects between these AAs and other nutrients or bioactive constituents naturally present in whole protein sources and the optimal ratios or combinations for mitigating EIGS in humans in exertional heat stress conditions are not yet fully understood.

In addition to the potential effects of AAs or whole proteins on EIGS, there is emerging evidence highlighting their capacity to contribute to whole‐body thermoregulation or perception of the heat. For example, isolated taurine supplementation (a semi‐essential sulphonic acid) improved local sweating responses during exercise in the heat, which delayed the rate of rise in core temperature (Page et al., 2019; Peel et al., 2024). Although l‐arginine has been associated with increased NO availability (Wu et al., 2021), it has not demonstrated an effect on sweating responses (Cable et al., 2024; Tyler et al., 2016). Furthermore, the effect of selected AAs on vascular function is another key area of interest. For example, l‐arginine was reported to improve flow‐mediated dilatation (FMD) across various populations (Bai et al., 2009; Ellis et al., 2016), but its effects on FMD in exercising or athletic populations are not well established. Furthermore, acute whey protein supplementation improved the FMD response among overweight (Ballard et al., 2013) and healthy adults (Oliveira et al., 2020), probably owing to the presence of bioactive peptides and greater branched‐chain amino acid (BCAA) content, enhancing endothelial function via endothelial NO synthase expression (Ballard et al., 2013). In contrast, fish protein hydrolysate lacks these effects, possibly owing to its lower content of bioactive peptides and BCAAs (Oliveira et al., 2020). This enhancement of vascular function offers a potential advantage to exercising humans in the heat, assuming that these effects are conferred to the subcutaneous vasculature, where dry heat losses can be increased (Périard et al., 2021; Simmons et al., 2011).

It is apparent that AAs or some animal‐derived whole protein sources have the potential to influence physiological function in the heat, but given the risk of EIGS in exercising athletes, it is important that pre‐exercise supplementation choices are also considerate of gut health. Plant‐based AAs have been promoted on this basis (Gorissen et al., 2018), which also aligns with growing trends in sustainable and health‐conscious nutrition (Gil et al., 2024; Gorissen et al., 2018). Indeed, in animal models, soy and pea proteins were reported to improve gut barrier integrity (Basson et al., 2021); however, consumption of some protein sources, such as whey protein, during exercise might lead to digestibility issues, with an increase in GIS observed previously (Snipe et al., 2017). The potential uses of plant‐based protein sources remain largely unexplored in the context of exercise‐induced gut damage and thermoregulation. This is important because plant foods, specifically pulses such as fava beans, are naturally rich in vitamins and minerals that provide numerous physiological benefits (Badjona et al., 2023; Labba et al., 2021; Multari et al., 2015). As such, legume protein isolate might support exercise tolerance in the heat by providing key minerals and bioactive peptides that help to reduce inflammation, promote beneficial gut bacteria and stimulate short‐chain fatty acid production to protect gut integrity (Gullón et al., 2015).

Although consumption of some isolated AAs might aid in reducing intestinal damage and mitigating associated EIGS during exercise in the heat, there has been no investigation of a multiple AA blend in an isolated plant‐protein source. The aim of this study was to investigate the effect of a novel fava bean plant‐based protein supplement on EIGS, thermoregulation and endurance performance during exercise in the heat. We hypothesized that fava bean plant‐based protein supplementation would attenuate biomarkers of intestinal damage and inflammation, reduce the incidence and severity of GIS and improve endurance performance in hot conditions in comparison to placebo.

## 2 MATERIALS AND METHODS

### 2.1 Participants

Twelve healthy males (*n* = 5) and females (*n* = 7) were recruited to take part in this study during autumn–winter months (September–December) in the UK to minimize heat acclimatization effects [31 ± 5 years of age; stature, 1.7 ± 0.1 m; body mass, 74.4 ± 15.0 kg; peak oxygen uptake (${\dot{V}}_{O_{2}\mathrm{peak}}$), 51.0 ± 5.1 mL/kg/min]. Participants were recreationally active (exercising moderately at least two times per week), self‐identified as having the capacity to run consistently for 1 h at a moderate‐to‐high intensity and were free from chronic diseases, GI issues or recent use of substances affecting GI integrity (e.g., prebiotics, probiotics or antibiotics). All participants completed all elements of the trial and provided written informed consent, and institutional ethical approval was provided for this study (2 2024 9972 9612), which was conducted in accordance with the 2013 *Declaration of Helsinki*.

### 2.2 Study design

**FIGURE 1:**
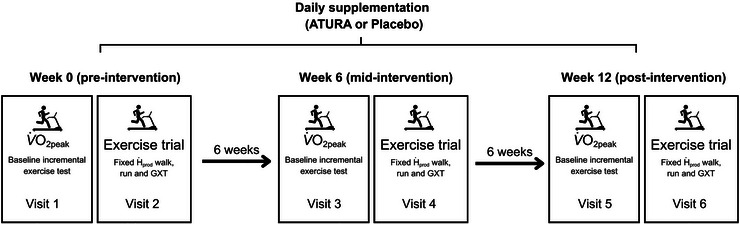
Schematic diagram of study design. Abbrevations: GXT, graded exercise test; $\dot{H}$_prod_, heat production; ${\dot{V}}_{O_{2}\mathrm{peak}}$, peak oxygen consumption.

**FIGURE 2:**
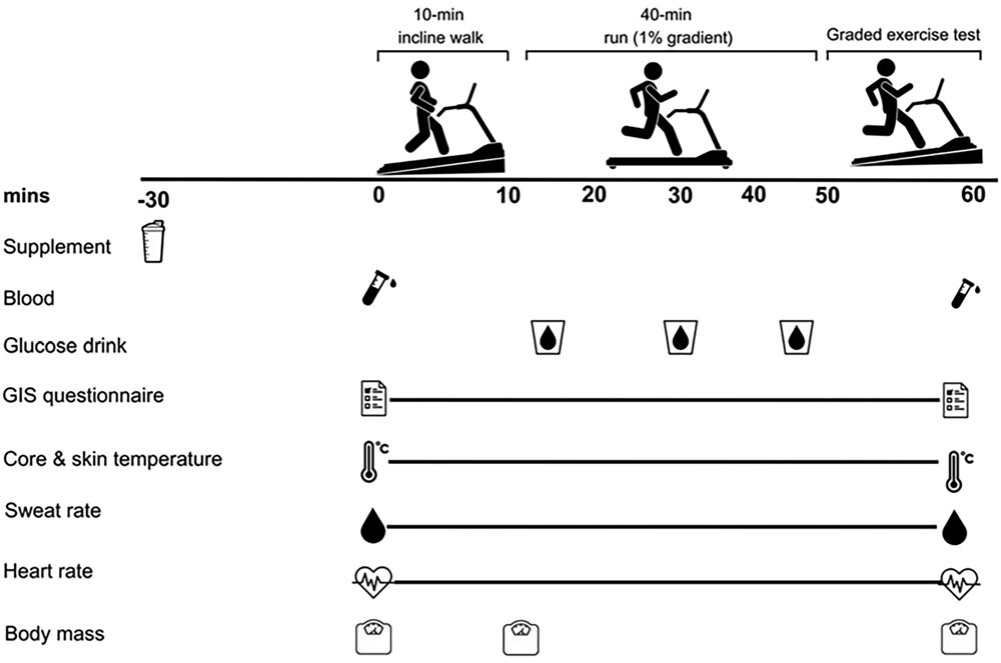
Schematic diagram of the exercise trial. Continuous black line represents continuous measurement. Abbrevation: GIS, gastrointestinal symptoms.

In a randomized, double‐blind, placebo‐controlled crossover trial, participants received either a plant‐based isolate of fava bean protein (ATURA; 60 g/day; ATURA, Deltagen, Bristol, UK) or a taste‐ and colour‐matched placebo (vanilla‐flavoured powder, Elite Sports Nutrition, 2024; orange food colourant, Roxy & Rich Inc., 2024). After prescreening (visit 1), participants completed a baseline visit (visits 2 and 4) and exercise trial (visits 3 and 5) (Figure 1). Participants completed a 3‐day weighed food diary during each intervention period to monitor dietary intake and ensure consistency throughout the study. During the exercise trial, participants completed a three‐stage exercise protocol in the heat (Figure 2). A minimum washout of 4 days and a maximum of 12 days were completed between study conditions (Brennan et al., 2019). All female participants were non‐users of hormonal contraceptives and completed both trials in the follicular phase of the menstrual cycle, determined using calendar‐based counting.

**FIGURE 3:**
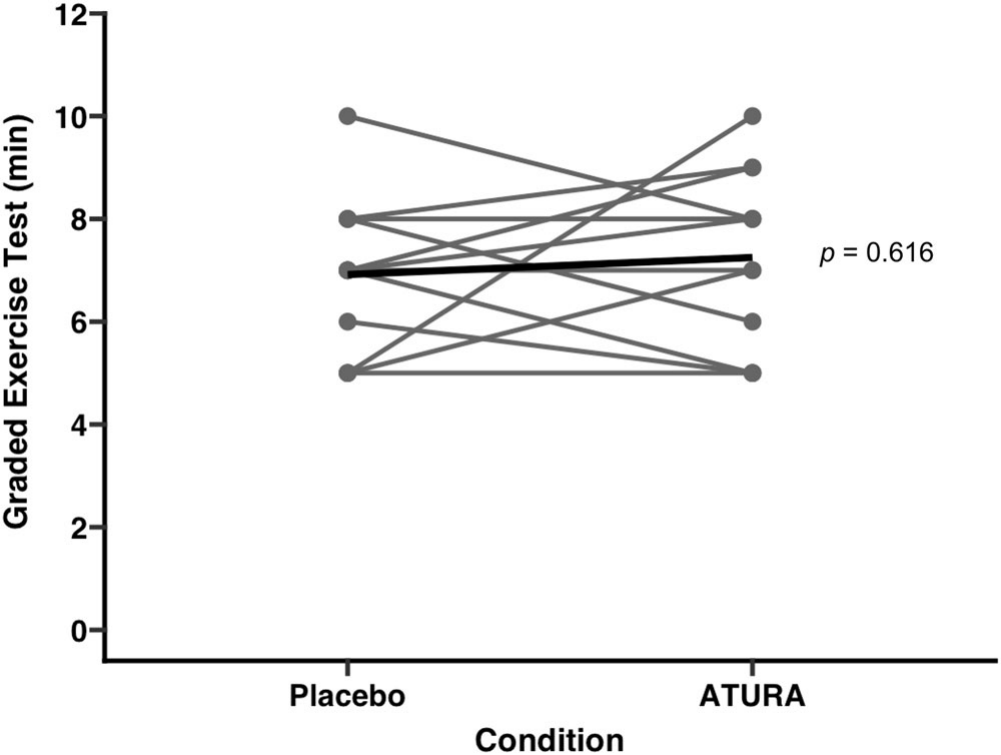
Graded exercise test following the fixed heat production walk and run, for ATURA and Placebo conditions. Each grey line represents one participant. Black bar denotes mean response (*P* = 0.616; *n* = 12).

### 2.3 Habitual dietary intake

Three‐day weighed food diaries were used to determine habitual nutrient intake, as detailed by Page et al. (2024). In brief, participants were asked to weigh and record all food and drink consumption on two weekdays and one weekend day during each supplement arm of the study. Scales were accurate to 0.1 g (Superior mini‐Digital Kitchen Scale, CHWARES, Guangzhou, China). All food diary analysis was conducted using an online dietary analysis software package (Nutritics, Research Edition, v.5.83, Dublin, Ireland).

### 2.4 Baseline incremental test

During visits 2 and 4, participants completed a modified incremental exercise test to exhaustion (Bruce et al., 1973) on a motorized treadmill (h/p/cosmos, Am Sportplatz 8, Nußdorf, Germany) to assess peak oxygen consumption (${\dot{V}}_{O_{2}\mathrm{peak}}$), using a breath‐by‐breath gas analyser (Jaeger Vyntus CPX, Hoechberg, Germany). Participants completed a standardized 5 min warm‐up at a walking pace of 5 km/h. The test then commenced at a speed of 5 km/h and incline of 1.5%, with increments of 1 km/h and 0.5% every 3 min. The test was terminated when participants reached volitional exhaustion (American College of Sports Medicine, 2014). The criterion for achieving exhaustion included a respiratory exchange ratio (RER) > 1.15, rating of perceived exertion (RPE) of 19–20 and maximal heart rate (HR) within 10 beats/min of age‐predicted maximum (220 − age; Poole et al., 2008). From the incremental exercise test, ${\dot{V}}_{O_{2}\mathrm{peak}}$ was obtained as the final 30 s average. The test was designed to increase mechanical work rate progressively to elicit a range of heat production (*Ḣ*_prod_) values, including that required for the treadmill walking protocol (6 W/kg of body mass) and running protocol (10 W/kg of body mass) in visits 3 and 5. The *Ḣ*_prod_ of each participant for the experimental trials was determined by subtracting the rate of mechanical work (Wk) from the rate of metabolic energy expenditure (*Ṁ*; Eq. 1).

(1) $${\dot{H}}_{\mathrm{prod}}=\dot{M}-\mathrm{Wk}\left(\mathrm{in}\;\mathrm{watts}\right)$$

where metabolic energy expenditure (*Ṁ*) was determined using measured oxygen consumption (${\dot{V}}_{O_{2}}$; in litres per minute) and RER within the final 1 min of each stage for the baseline incremental test (Eq. 2):

(2) $$\begin{array}{ccc} & & \dot{M}={\dot{V}}_{o_{2}}\times \;\frac{\left(\left(\;\frac{\mathrm{RER}-0.7}{0.3}\;\right)\;\times \;21.13\right)+\left(\left(\;\frac{1.0-\mathrm{RER}}{0.3}\;\right)\;\times \;19.62\right)}{60}\\ & & \times \;1000\;\left(\mathrm{in}\;\mathrm{watts}\right) \end{array}$$

The $\dot{H}$_prod_ (in watts per metre squared) was expressed relative to the body surface area (BSA) of participants (Cramer and Jay 2019; Du Bois and Du Bois, 1916):

(3) $${\dot{H}}_{\mathrm{prod}}=\frac{{\dot{H}}_{\mathrm{prod}}}{\mathrm{BSA}}\;\left(\mathrm{in}\;\mathrm{watts}\;\mathrm{per}\;\mathrm{metre}\;\mathrm{squared}\right)$$

(4) $$\begin{array}{ccc} & & \mathrm{BSA}=0.00718\times \left[\mathrm{body}\mathrm{mass}{\left(\mathrm{kg}\right)}^{0.425}\right]\times \left[\mathrm{height}{\left(\mathrm{cm}\right)}^{0.725}\right]\\ & & \left(\mathrm{in}\mathrm{metres}\mathrm{squared}\right) \end{array}$$

### 2.5 Experimental trials

#### 2.5.1 Pretrial instrumentation

Participants arrived hydrated after consuming a self‐selected meal of low‐fermentable oligosaccharides, disaccharides, monosaccharides and polyols, as recommended by Costa et al. (2022), with euhydration confirmed by urine osmolality of <600 mosmol/kg H_2_O using an osmometer (Osmocheck, Vitech Scientific Ltd, UK). Prior to the exercise trial (30 min; Kozior et al., 2023), participants consumed a standardized drink (ATURA or Placebo). Participants wore standardized running shorts, socks and trainers, with a sports bra for female participants. Participants self‐inserted a flexible rectal probe (Walters Medical, St Albans, UK) 10 cm past the anal sphincter in a private area and under prior instructions from the researcher. Core temperature was continuously recorded using a data logger (SQ2010; Grant Instruments Ltd, Cambridge, UK). Participants were also instrumented with an HR monitor (Polar Heart Rate Monitor M400, Warwick, UK), which was monitored continuously throughout each exercise trial. Venous (30 mL) and capillary blood samples from fingerpick (in duplicate, after 5 min seated rest) were collected immediately before the trial.

#### 2.5.2 Exercise trial

Participants entered the environmental chamber (35 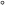 C dry‐bulb temperature, 40% relative humidity) and were instrumented with skin thermistors (Grant Instruments Ltd, Cambridge, UK) at four sites (upper chest, mid‐humerus, mid‐calf and mid‐thigh). Ramanathan's equation (Ramanathan, 1964) was used to calculate weighted mean skin temperature. A ventilated sweat capsule system (Q‐Sweat; WR Medical Electronics Co., Stillwater, MN, USA) was fitted to the participant's back to measure local sweating rate (LSR). Pulmonary ${\dot{V}}_{O_{2}}$ was measured continuously via a calibrated breath‐by‐breath gas analyser (Jaeger Vyntus CPX, Hoechberg, Germany). Participants walked at an incline on a motorized treadmill (6 W/kg of body mass) for 10 min to control $\dot{H}$_prod_, which was based upon the relationship between metabolic $\dot{H}$_prod_ and treadmill work rate in the baseline incremental test. At 10 min, participants transitioned immediately into a run for 40 min (10 W/kg of body mass). This was immediately followed by a graded exercise test (GXT) to assess exercise tolerance in the heat, which began at the participant's treadmill speed at the end of the second stage and was incremented by 1 km/h every 1 min until volitional exhaustion. Environmental conditions, such as ambient dry‐bulb temperature (in degrees Celsius), relative humidity and air velocity (in metres per second), were monitored continuously next to the participant (Kestrel 5400 Heat Stress Tracker, Kestrel Meters, Boothwyn, PA, USA). An electric fan (SIP 24″ Drum Fan, Loughborough, UK) was placed in front of the participant during the exercise period on the treadmill, providing an airflow 1 m/s directed at the torso. During the trial, HR and breath‐by‐breath gas samples were measured continuously, and RPE, thermal comfort, thermal sensation and GIS were recorded every 5 min. The RPE was recorded using a 6‐ to 20‐point Borg scale (Borg, 1982), and thermal comfort was recorded using a 7‐point scale (where −3 = ‘much too cool’, 0 = ‘comfortable’ and 3 = ‘much too warm’; Bedford, 1936). Thermal sensation was recorded using a 9‐point scale (where −4 = ‘very cold’, 0 = ‘neutral’ and 4 = ‘very hot’; Zhang et al., 2004). GI symptoms were recorded on an 11‐point scale (where 0 = no symptoms, 1–4 = mild symptoms, 5–9 = severe symptoms and 10 = extremely severe symptoms; Gaskell et al., 2019). Participants consumed 200 mL carbohydrate solution (63 g dextrose, Myprotein) during the trial (10, 30 and 50 min) to place additional demand on the gut during exercise, whilst simulating real‐world endurance exercise conditions, where carbohydrate feeding is common but often linked to gut distress (Costa et al., 2017). The body mass of each participant was measured (whilst wearing running shorts, a sports bra for females, the HR monitor and the inserted rectal thermistor) using a calibrated scale (resolution 50 g; Seca 711, Hamburg, Germany). Immediately postexercise, venous (30 mL) blood samples and after a seated 5 min rest, capillary blood samples, in duplicate, were collected, followed by a venous blood sample (30 mL) 1 h postexercise (Figure 2).

### 2.6 Supplements

Participants consumed ATURA Fava Bean Protein (ATURA; 60g/day; ATURA, Deltagen, Bristol, UK) or a placebo. The 8 day loading protocol followed established AA supplementation protocols (Costa et al., 2023; Zuhl et al., 2014) to achieve physiological AA elevations while allowing GI adaptation prior to exercise testing. The 60 g/day dose (2 × 30 g servings) delivered AA concentrations comparable to commercial protein supplements (Gorissen et al., 2018) and a previous AA study observing a significant reduction in postexercise i‐FABP (Costa et al., 2023), while minimizing total protein load to reduce potential GI discomfort during subsequent exercise (Snipe et al., 2017). To ensure blinding, both drinks contained vanilla‐flavoured powder (3 g; designer flavoured powder, Elite Sports Nutrition, UK) and an orange water‐soluble food colourant (Roxy & Rich Inc., Canada). Participants were provided with 14 opaque bottles, each containing the desired ATURA or placebo, consumed with water. Participants consumed two 30 g servings per day, in the morning and afternoon/evening. All 14 bottles were returned empty by participants, and daily logs were tracked for compliance (100% compliance). The AA composition of the ATURA (Table 1) was conducted by Deltagen UK Ltd. The analysis was performed as batch analysis, and all participants received the same batch of ATURA. No participants experienced any GI disturbance during the supplementation period, assessed by the gastrointestinal symptom rating scale (Bovenschen et al., 2006). A post‐trial blinding assessment questionnaire confirmed effective blinding, with 80% of participants unable to distinguish supplement conditions. The ATURA supplements do not contain any of the prohibited substances, nor is it manufactured using any prohibited methods listed in the ‘World Anti‐Doping Code’ (International Standard) Prohibited List January 2023, confirmed at Deltagen UK Ltd.

**TABLE 1:** Amino acid composition for each day (60 g) of ATURA.

Amino acid	ATURA	
	Per 30 g serving	Per 60 g daily serving
Alanine	0.94	1.88
Arginine	2.15	4.30
Aspartic acid	2.75	5.50
Glutamic acid	4.14	8.28
Glycine	0.95	1.91
Histidine	0.62	1.25
Isoleucine	1.04	2.09
Leucine	1.94	3.89
Lysine	1.55	3.11
Methionine	0.15	0.31
Phenylalanine	1.10	2.20
Proline	1.04	2.08
Serine	1.22	2.44
Threonine	0.80	1.60
Tryptophan	0.20	0.40
Tyrosine	0.83	1.66
Valine	1.13	2.27
Cysteine	0.23	0.46

*Note*: Amino acid concentrations are presented in grams.

### 2.7 Vascular function measurements

At the baseline visit following an overnight fast, vascular function was assessed non‐invasively through FMD ultrasound of the brachial artery, in accordance with established guidelines (Thijssen et al., 2019) and as previously described (John et al., 2024). Upon arrival at the laboratory, participants rested in the supine position on a plinth for 15 min in a dark, quiet and temperature‐controlled room. After the resting period, blood pressure was measured in the participant's left arm. B‐Mode ultrasonography (Esaote, MyLab9, Genoa, Italy) was used to identify the brachial artery in the right arm via B‐mode echo and pulse‐wave Doppler velocity. An ultrasound probe was placed longitudinally on the brachial artery proximal to the antecubital fossa, with a pressure cuff (Hokanson SC5 Vascular Tourniquet) wrapped distal to the probe (3–5 cm) on the forearm. Once comfortable, the test commenced using automated edge‐detection software (FMD Studio, Cardiovascular Suite, Quipu, Italy). The protocol consisted of a 1 min baseline artery diameter recording, 5 min of cuff inflation at 220 mmHg, and a 4 min post‐dilatation recording to assess the change in arterial diameter in response to increased shear stress, with a total measurement duration of 10 min.

### 2.8 Partitional calorimetry

Heat balance parameters, such as *Ḣ*_prod_, evaporative requirement for heat balance (*Ė*_req_), evaporation at the skin surface (*Ė*_skin_) and heat storage were estimated via partitional calorimetry (Cramer and Jay 2019; Du Bois & Du Bois, 1916).

On the assumption that blood entering and leaving the cutaneous circulation was equal to core temperature (*T*_core_) and skin temperature (*T*_skin_), respectively, maximum skin blood flow (SkBF) was determined as follows (Sawka & Young, 2006):

(5) $$\mathrm{SkBF}\;=\frac{\left(\frac{1}{\mathrm{SH}}\;\times \;{\dot{H}}_{\mathrm{prod}}\right)}{\left(T_{\mathrm{core}\;}-\;T_{\mathrm{skin}}\right)}$$

where SH is specific heat of the blood (∼1 kcal/°C) and $\dot{H}$_prod_ is expressed in kilocalories per minute.

(6) $${\dot{E}}_{\mathrm{req}}={\dot{H}}_{\mathrm{prod}}-{\dot{H}}_{\mathrm{dry}\;\mathrm{skin}}-{\dot{H}}_{\mathrm{res}}\left(\mathrm{in}\;\mathrm{watts}\right)$$

where $\dot{H}$_dry skin_ is dry heat exchange at the skin surface and $\dot{H}$_res_ is respiratory heat loss.

(7) $${\dot{H}}_{\mathrm{dry}\mathrm{skin}}=C_{\mathrm{skin}}+R_{\mathrm{skin}}+K_{\mathrm{skin}}\left(\mathrm{in}\;\mathrm{watts}\right)$$

where *C*_skin_ is convection, *R*_skin_ is radiation and *K*_skin_ is conduction.

(8) $$C_{\mathrm{skin}}+R_{\mathrm{skin}}=\frac{\left(T_{\mathrm{skin}}-\;\mathrm{to}\right)}{\left(R\mathrm{cl}\frac{1}{h\times cl}\right)}\times \mathrm{AD}$$

where *T*_skin_ is skin temperature (equation 21); to, operative temperature; *R*_cl_, dry heat transfer of clothing; *h*, combined convective heat transfer coefficient; *f*_cl_, clothing area factor; and AD, body surface area.

(9) $$\mathrm{to}\;=\frac{h_{r}t_{r}+h_{c}t_{a}\;}{h_{r}+h_{c}}\;\left(\mathrm{in}\;\mathrm{watts}\right)$$

where *h*_r_ is radiative heat transfer coefficient; *t*_r_, radiant temperature; *h*_c_, convective heat transfer coefficient; and *t*_a_, ambient air temperature.

(10) $$h=h_{c}+h_{r}\left(\mathrm{in}\mathrm{watts}\mathrm{per}\mathrm{metre}\mathrm{squared}\mathrm{per}\mathrm{kelvin}\right)$$

(11) $$h_{c}=8.3\times \frac{0.6}{V_{\mathrm{air}}}\left(\mathrm{in}\mathrm{watts}\mathrm{per}\mathrm{metre}\mathrm{squared}\mathrm{per}\mathrm{kelvin}\right)$$

where *V*_air_ is ambient air velocity.

(12) $$\begin{array}{ccc} h_{r} & = & 4\varepsilon \sigma \frac{A_{r}}{A_{D}}{\left(273.2+\frac{T_{\mathrm{skin}}+t_{r}}{2}\right)}^{3}\\ & & \left(\mathrm{in}\mathrm{watts}\mathrm{per}\mathrm{metre}\mathrm{squared}\mathrm{per}\mathrm{kelvin}\right) \end{array}$$

where ε is non‐dimensional emissivity of the body surface; σ, Stefan–Boltzmann constant; *A*_r_/*A*_D_, fraction of the body surface participating in radiative heat transfer.

(13) $$H_{\mathrm{res}}=C_{\mathrm{res}}+E_{\mathrm{res}}\left(\mathrm{in}\mathrm{watts}\right)$$

where *C*_res_ is convective respiratory heat loss and *E*_res_ is evaporative respiratory heat loss.

(14) $$C_{\mathrm{res}}=0.001516\times \dot{M}\;\left(28.56+0.641\times P_{a}\;-0.885\times t_{a}\right)\left(\mathrm{in}\;\mathrm{watts}\right)$$

where *Ṁ* is metabolic energy expenditure and *P*_a_ is vapour pressure of inspired air.

(15) $$P_{a}=\frac{6.116441\times 10\left(\frac{7.5911386\times t_{a}}{t_{a}+240.7263}\right)\times \frac{\%\mathrm{RH}}{100}}{10}$$

where %RH is relative humidity.

(16) $${\dot{E}}_{\mathrm{skin}}=\mathrm{delta}\mathrm{body}\mathrm{mass}\mathrm{loss}\times \frac{\lambda }{1000}\left(\mathrm{in}\mathrm{kilojoules}\right)$$

where λ is the latent heat of vaporization of sweat (2426 J/g).

(17) $$\begin{array}{ccc} & & \mathrm{Heat}\;\mathrm{storage}\;=\;\mathrm{time}\;\times \;\frac{\dot{H}\mathrm{prod}-\;\dot{H}\mathrm{dry}\;\mathrm{skin}-\;\dot{H}\mathrm{evap}\;-\;\dot{H}\mathrm{res}\;}{1000}\\ & & \left(\mathrm{in}\;\mathrm{kilojoules}\right) \end{array}$$

(18) $${\dot{H}}_{\mathrm{evap}\mathrm{skin}}=\mathrm{WBSR}\times \lambda \times \frac{\mathrm{Seff}}{60}\left(\mathrm{in}\mathrm{watts}\right)$$

where WBSR is whole‐body sweat rate, based on body mass changes over time (in grams per minute).

(19) $$\mathrm{Seff}\;=1\;-\;\frac{\omega \mathrm{req}2}{2}\;\left(\mathrm{ND}\right)$$

(20) $$\omega_{\mathrm{req}}=\frac{{\dot{E}}_{\mathrm{req}}}{{\dot{E}}_{\max}}$$

(21) $$T_{\mathrm{skin}}=\left(T_{\mathrm{chest}}+T_{\mathrm{arm}}\right)\times 0.3+\left(T_{\mathrm{thigh}}+T_{\mathrm{calf}}\right)\times 0.2\left(\mathrm{in}\mathrm{degrees}\mathrm{Celsius}\right)$$

(22) $$PM_{\mathrm{post},c}=PM_{\mathrm{post},u}\times \left(1+\Delta \mathrm{PV}\right)$$

where PM_post,c_ and PM_post,u_ indicate corrected and uncorrected serum or plasma biomarker postexercise, respectively, and PV is plasma volume.

### 2.9 Blood sampling

Blood was also sampled into capillary tubes and microcuvettes (Hemocue Hb 201) for the measurement of haematocrit and haemoglobin concentration, respectively, to estimate plasma volume (PV) changes (Dill & Costill, 1974). The capillary tubes were spun in a microcentrifuge (Hawksley Neuation HCT Haematocrit Centrifuge, iFuge‐HCT, Hawksley & Sons Ltd, UK) at 4,500 *g* for 5 min, and separated red cell volume was measured using a haematocrit reader (Hawksley Micro‐Haematocrit Reader). All samples were obtained and measured in duplicate, with the mean value recorded for analysis. Venous blood samples were obtained at each visit (Figure 1) via venipuncture from an antecubital vein into two EDTA‐treated vacutainer tubes and two untreated EDTA tubes. These tubes were kept at room temperature for 20 min and in ice for 15 min until centrifugation of 2800*g* for 10 min at 4°C (Heraeus Labofuge 400 Centrifuge, Model: 400/400R, Thermo Fisher Scientific, Waltham, MA, USA). The plasma and serum were pipetted into 1.5 mL cryotubes and stored in a −80°C freezer for later analysis.

### 2.10 Blood analysis

Concentrations of i‐FABP (marker of enterocyte damage) and sCD14 (marker of gut barrier dysfunction) from EDTA plasma and IL‐6 and HSP70 (marker of cellular stress) from serum were assessed via ELISA using commercially available kits (Hycult Biotechnology, Uden, The Netherlands for i‐FABP and sCD14; R&D Systems, Minneapolis, MN, USA for IL‐6 and HSP70), according to the manufacturers’ instructions. EDTA plasma and serum samples were thawed at room temperature and diluted in assay buffer at 2× for i‐FABP, 80× for sCD14 and 2× for both IL‐6 and HSP70. Absorbance was measured spectrophotometrically using a FLUOstar Omega microplate reader (BMG LABTECH, Ortenberg, Germany) at the wavelengths specified by the manufacturers. Assay ranges were as follows: i‐FABP, 47–3000 pg/mL; sCD14, 8.8–100 ng/mL; IL‐6, 9.4–600 pg/mL; and HSP70, 125–8000 pg/mL. The intra‐assay coefficient of variation for between‐sample duplicates was <10% for all analytes. Data were analysed using four‐parameter logistic regression to calculate analyte concentrations based on the standard curve, with the mean of duplicates used for final analysis, and concentrations were adjusted for PV changes (Equation 22; Matomäki et al., 2018).

### 2.11 Statistical analysis

To determine an a priori sample size, an effect size of *d* = 0.89 was considered, based on previous research to detect a change in postexercise circulating i‐FABP (Morrison et al., 2014), with 80% power at the α‐level of 0.05 using G*Power (v.3.1.9.7; Universität Düsseldorf, Germany). This determined that a sample size of 12 was required to observe differences between groups. Circulating i‐FABP was regarded as the primary outcome; all other physiological, thermoregulatory, performance, perceptual, GI and vascular measures were classified as secondary/exploratory outcomes. No multiplicity correction was applied across secondary/exploratory outcomes; these are interpreted with appropriate caution and positioned as hypothesis‐generating rather than confirmatory. The normality of the residuals was assessed using the Shapiro–Wilk test, after which two‐way ANOVAs were used to determine the effect of condition (ATURA vs. Placebo) and time (0–50 min) on ${\dot{V}}_{O_{2}}$, HR, RER, $\dot{H}$_prod_, *T*_core_ and *T*_skin_ using the base R stats package. For trial sections (fixed $\dot{H}$_prod_ walk or run), a two‐way ANOVA for condition effects by trial section was used for outcomes of partitional calorimetry. Student's wo‐tailed paired *t*‐tests were used to compare conditions (ATURA vs. Placebo) for GXT time, ${\dot{V}}_{O_{2}\mathrm{peak}}$ at visits 1 and 3 and ${\dot{V}}_{O_{2}\mathrm{peak}}$ during the exercise trial, in addition to WBSR, SkBF and the weighed food diary. GIS were analysed using Friedman's test. Partitional calorimetry outcomes ($\dot{H}$_prod_, $\dot{E}$_skin_, heat storage, $\dot{E}$_req_ and dry heat loss, ${\dot{H}}_{\mathrm{dry}\mathrm{skin}}$) were analysed using a repeated‐measures ANOVA with fixed effects for condition (ATURA vs. Placebo) and trial section (walk vs. run), including the condition × trial section interaction term. For significant main effects or interactions, *post hoc* pairwise comparisons with Bonferroni adjustment were performed to identify specific differences between conditions and/or trial sections. Significant main and interaction effects were analysed using the emmeans package, with Bonferroni‐corrected *post hoc* tests used to determine pairwise differences. Thermal comfort and thermal sensation analysis demonstrated consistent non‐normal distribution of residuals; therefore, analysis was conducted using the non‐parametric aligned ranks test for repeated measures (‘*ARTool*’ package in RStudio). Trial order effects were assessed using Student's paired *t*‐tests. An α‐level of 0.05 was set a priori for all statistical tests. All data collected were analysed using R (R Core Team, 2020) in RStudio (Rstudio Team, 2020). Partial eta‐squared (η_p_^2^) was reported to calculate the magnitude of effect according to the following criteria: 0.02 = a small difference, 0.13 = a moderate difference and 0.26 = large difference (Cohen, 1988). Cohen's *d* for repeated measures was calculated as the mean difference divided by the SD of the differences to interpret the effect of pairwise changes. Cohen's threshold effect sizes were classified as small (*d* = 0.2), medium (*d* = 0.5) and large (*d* ≥ 0.8), with 95% confidence intervals (95% CI) reported for all effect sizes.

## 3 RESULTS

### 3.1 Dietary responses

For the three‐day weighed food diary, between‐condition differences were assessed using Student's two‐tailed paired *t*‐tests. There were no significant differences between supplement groups for total calorie intake [*t*_(11)_ = 1.68, *P* = 0.121], carbohydrate [*t*_(11)_ = 1.63, *P* = 0.130], fat [*t*_(11)_ = 1.69, *P* = 0.118] or protein [*t*_(11)_ = 0.03, *P* = 0.973]. Moreover, no significant differences were observed for intake of individual AAs (all *P* > 0.05; see Supplementary File 1).

### 3.2 Exercise responses

Exercise responses were analysed using two‐tailed paired‐samples *t*‐tests to compare conditions (ATURA vs. Placebo). There were no differences in GXT time between the conditions {*t*_(11)_ = −0.511, *P* = 0.616, Cohen's *d* = 0.22, 95% CI [−1.70, 1.03]; Figure 3}. There was no difference in ${\dot{V}}_{O_{2}\mathrm{peak}}$ during the exercise trial between conditions {ATURA = 43.3 ± 6.5 mL/kg/min; Placebo = 44.5 ± 6.6 mL/kg/min; *t*_(11)_ = 0.422, *P* = 0.681, Cohen's *d* = 0.18, 95% CI [−4.58, 6.86]}. Additionally, baseline ${\dot{V}}_{O_{2}\mathrm{peak}}$ on visits 1 and 3 was not different between conditions {ATURA = 49.4 ± 5.8 mL/kg/min; Placebo = 50.2 ± 4.7 mL/kg/min; *t*_(11)_ = 0.391, *P* = 0.699, Cohen's *d* = 0.17, 95% CI [−3.78, 5.53]}. There was a trial order effect for ${\dot{V}}_{O_{2}\mathrm{peak}}$ during the heat exercise trial, which increased from trial 1 and 2 (*P* = 0.020), with no trial order effect for baseline ${\dot{V}}_{O_{2}\mathrm{peak}}$ (*P* = 0.500) and GXT time (*P* = 0.282).

### 3.3 Cardiometabolic responses

**FIGURE 4:**
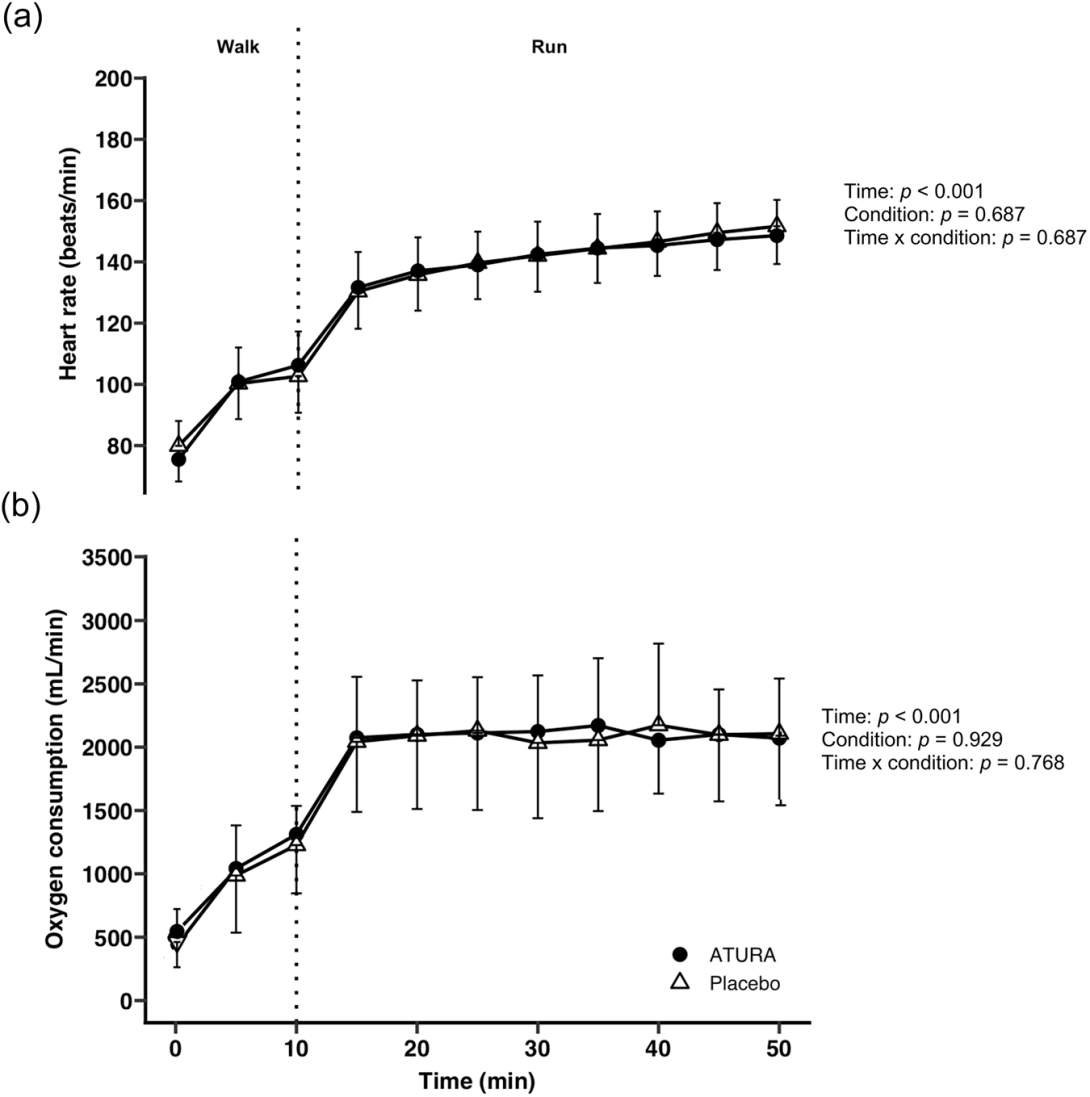
Heart rate (a) and oxygen consumption (b) following the fixed heat production walk and run for ATURA or Placebo conditions. No condition or interaction effect was observed for heart rate (condition, *P* = 0.687; interaction, *P* = 0.379) or oxygen consumption (condition, *P* = 0.929; interaction, *P* = 0.768). *n* = 12.

Cardiometabolic responses during the exercise trial were analysed using two‐way repeated‐measures ANOVA, with condition (ATURA and Placebo) and time as within‐subject factors for HR, ${\dot{V}}_{O_{2}}$ and RER. Heart rate increased with time across both conditions [*F*_(1,11)_ = 991.679, *P* < 0.001, η_p_^2^ = 0.991; Figure 4a] and was similar between trials {Placebo vs. ATURA: 103 [95, 110] vs. 106 [99, 113] beats/min end‐walk; 152 [143, 160] vs. 149 [141, 156] beats/min end‐run}. No main effect of condition [*F*_(1,236)_ = 0.162, *P* = 0.687, η_p_^2^ = 0.050] or interaction [*F*_(1,236)_ = 0.112, *P* = 0.379, η_p_^2^ = 0.082] was observed. Oxygen consumption increased with time across both conditions [*F*_(1,11)_ = 394.771, *P* < 0.001, η_p_^2^ = 0.151] and was similar between trials {Placebo vs. ATURA: 1997 ± 535 [1638, 2357] vs. 2062 ± 459 [1771, 2354] mL/min end‐walk; 2556 ± 649 [2120, 2993] vs. 2694 ± 610 [2306, 3081] mL/min end‐run}. There was no main effect of condition [*F*_(1,223)_ = 0.008, *P* = 0.929, η_p_^2^ = 0.110] or interaction [*F*_(1,223)_ = 0.087, *P* = 0.768, η_p_^2^ = 0.073; Figure 4b]. RER increased with time across both conditions [*F*_(1,11)_ = 74.965, *P* < 0.001, η_p_^2^ = 0.891] and was similar between trials {Placebo vs. ATURA: 0.85 ± 0.03 [0.833, 0.87] vs. 0.87 ± 0.07 [0.825, 0.913] end‐walk; 0.87 ± 0.05 [0.863, 0.930] vs. 0.91 ± 0.09 [0.850, 0.96] end‐run}, with no main effect of condition [*F*_(1,236)_ = 2.019, *P* = 0.157, η_p_^2^ = 0.022] or interaction [*F*_(1,236)_ = 0.657, *P* = 0.419, η_p_^2^ = 0.921] observed. There were no trial order effects for HR (*P* = 0.532), oxygen consumption (*P* = 0.615) and RER (*P* = 0.173). Furthermore, Student's paired‐samples *t*‐test demonstrated that PV change did not differ between conditions {ATURA, −9.85% ± 4.31%; Placebo, −9.35% ± 4.31%; *t*_(11)_ = −1.3146, *P* = 0.215, Cohen's *d* = 0.50, 95% CI [−3.68, 1.01]}.

### 3.4 GI responses

GI symptoms (Table 2) were not different between conditions for total GIS [χ^2^_(1)_ = 1.291, *P* = 0.257], upper GIS [χ^2^_(1)_ = 1.221, *P* = 0.654] and lower GIS [χ^2^_(1)_ = 2.001, *P* = 0.158]. Likewise, no differences were found between conditions for individual symptoms, including abdominal sloshing (*P* = 0.317), abdominal pain (*P* = 1.00), bloating (*P* = 0.157), burping (*P* = 0.564), fluctuance (*P* = 0.157) and nausea (*P* = 0.317).

**TABLE 2:** Incidence of gastrointestinal symptoms of total, upper and lower gastrointestinal symptoms.

Symptom	ATURA		Placebo	
	Incidence {% [95% CI]}	Severity^a^	Incidence {% [95% CI]}	Severity^a^
**Total GIS**	39 [17, 67]	29 (0–8)	55 [28, 80]	34 (0–11)
**Upper GIS**	31 [12, 61]	23 (0–6)	35 [14, 66]	24 (0–8)
Bloating	8 [1, 33]	6 (0–2)	11 [2, 42]	10 (0–2)
Upper abdominal pain	4 [0, 27]	3 (0–1)	9 [1, 40]	3 (0–1)
Burping	19 [6, 40]	14 (0–3)	15 [4, 35]	11 (0–2)
Urge to vomit	0 [0, 24]	0 (0–0)	0 [0, 25]	0 (0–0)
**Lower GIS**	8 [1, 33]	6 (0–2)	20 [7, 49]	10 (0–3)
Flatulence	4 [0, 28]	3 (0–1)	9 [1, 40]	7 (0–2)
Urge to defecate	0 [0, 25]	0 (0–0)	0 [0, 25]	0 (0–0)
Abdominal sloshing	4 [0, 28]	3 (0–1)	4 [0, 28]	3 (0–1)
**Other**				
Nausea	5 [1, 30]	4 (0–1)	4 [0, 28]	3 (0–1)

*Note*: Total GIS, *P* = 0.257; upper GIS, *P* = 0.654; lower GIS, *P* = 0.158; abdominal sloshing, *P* = 0.317; abdominal pain, *P* = 1.00; bloating, *P* = 0.157; burping, *P* = 0.564; fluctuance, *P* = 0.157; nausea, *P* = 0.317.

Abbreviation: GIS, gastrointestinal symptoms.

^a^ Mean of summative accumulation and range of participants reporting incidence.

### 3.5 Circulating biomarkers

**FIGURE 5:**
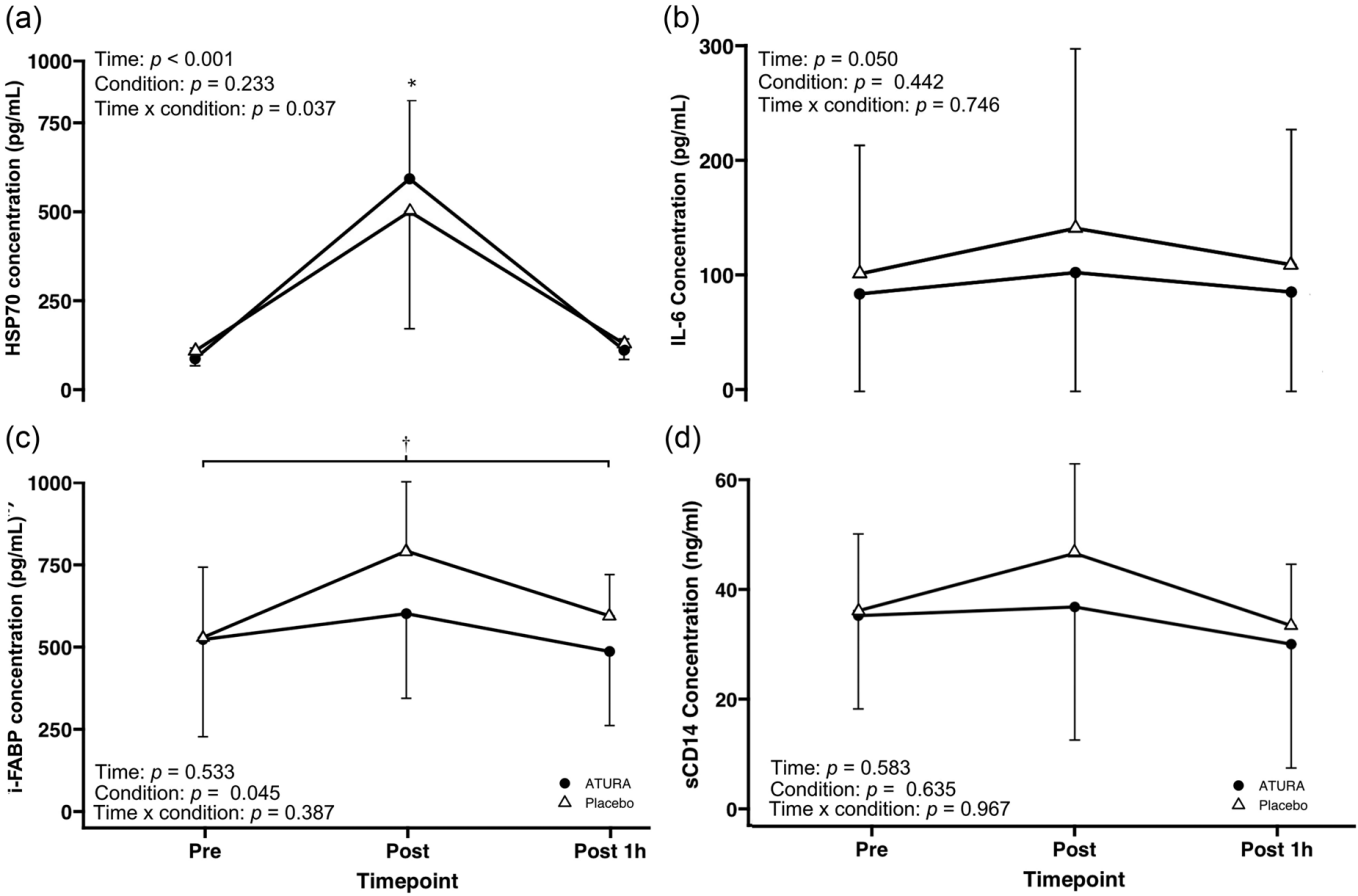
Circulating biomarkers for pre, post and 1 h post response to a fixed heat production walk, run and graded exercise test. Biomarkers include heat shock protein 70 (HSP70; a), interleukin‐6 (IL‐6; b), intestinal fatty acid‐binding protein (i‐FABP; c), and soluble claudin‐14 (sCD14; d). HSP70 (condition, *P* = 0.233; interaction, *P* = 0.037), IL‐6 (condition, *P* = 0.442; interaction, *P* = 0.746), i‐FABP (condition, *P* = 0.045; interaction, *P* = 0.387; time point, *P* = 0.533) and sCD14 (condition, *P* = 0.635; interaction, *P* = 0.967). Data are presented as the mean ± SD. *Significant postexercise difference between conditions (*P* < 0.05). †Significant main effect of condition (*P* < 0.05).

Circulating HSP70 increased from pre‐ to postexercise [*F*_(1,63)_ = 23.082, *P* < 0.001, η_p_^2^ = 0.687], with an interaction [*F*_(1,63)_ = 3.657, *P* = 0.037, η_p_^2^ = 0.152; Figure 5a] but no condition effect [*F*_(1,63)_ = 3.947, *P* = 0.233, η_p_^2^ = 0.061]; postexercise Placebo, [297, 703] pg/mL; ATURA, [373, 806] pg/mL [*post hoc*: *t*_(63)_ = −2.03, *P* = 0.047, *d* = 0.94]. Circulating IL‐6 increased pre‐ to postexercise [*F*_(1,63)_ = 4.597, *P* = 0.050, η_p_^2^ = 0.295], with no condition [*F*_(1,63)_ = 0.612, *P* = 0.442] or interaction effects [*F*_(1,63)_ = 0.108, *P* = 0.746, η_p_^2^ = 0.221; Figure 5b]; postexercise Placebo, [43, 241] pg/mL; ATURA, [5, 201] pg/mL. Circulating i‐FABP showed condition effect [*F*_(1,63)_ = 3.506, *P* = 0.045, η_p_^2^ = 0.120], no interaction [*F*_(1,63)_ = 0.963, *P* = 0.387] or time effects [*F*_(2,63)_ = 0.635, *P* = 0.533; Figure 5c]; postexercise Placebo, [654, 921] pg/mL; ATURA, [434, 760] pg/mL. Circulating sCD14 showed no condition [*F*_(1,63)_ = 0.227, *P* = 0.635], time point [*F*_(2,63)_ = 0.543, *P* = 0.583] or interaction effects [*F*_(2,63)_ = 0.033, *P* = 0.967; Figure 5d]. There were no trial order effects (*P* > 0.05).

### 3.6 Thermoregulatory responses

**FIGURE 6:**
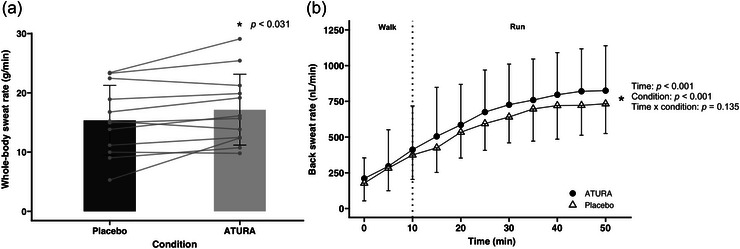
Whole‐body sweat rate (a) during the fixed heat production walk, run and GXT and back local sweat rate (b) following the fixed heat production walk and run for both conditions. Whole‐body sweat rate (*P* = 0.031), back local sweat rate (condition, *P* < 0.001; interaction, *P* = 0.135). *Significant condition effect (*P* < 0.05; *n* = 12).

WBSR was compared using Student's two‐tailed paired‐samples *t*‐test and increased in the ATURA conditions compared with Placebo {*t*_(11)_ = 2.487, *P* = 0.031, Cohen's *d* = 0.72, 95% CI [0.21, 3.39] g/min; Figure 6a}. Back LSR, indicated by a two‐way repeated‐measures ANOVA, increased with time across both conditions [*F*_(1,11)_ = 102.347, *P* < 0.001, η_p_^2^ = 0.931] and was greater in the ATURA compared with Placebo conditions, as indicated by a condition effect for back LSR [*F*_(1,227)_ = 22.376, *P* < 0.001, η_p_^2^ = 0.090; Figure 6b], with no interaction effect [*F*_(1,227)_ = 2.764, *P* = 0.135, η_p_^2^ = 0.073; Figure 6b]. At the end of the walk, back LSR was 375 ± 171 [260, 490] nL/min for Placebo and 412 ± 306 [218, 606] nL/min for ATURA; at the end of the run, it was 733 ± 208 [593, 873] nL/min for Placebo and 825 ± 314 [625, 1025] nL/min for ATURA. There were no trial order effects for WBSR (*P* = 0.165) and back LSR (*P* = 0.339).

**FIGURE 7:**
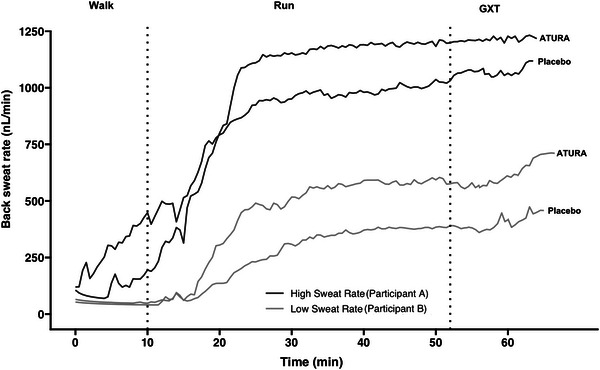
Representative traces of high sweat rate (Participant A) and low sweat rate (Participant B) from back sweat rate during the fixed $\dot{H}$_prod_ walk, run and GXT. Two individual participants for each condition (*n* = 2). Abbrevations: GXT, graded exercise test; $\dot{H}$_prod_, heat production.

Figure 7 presents representative traces of back LSR between two individual participants with a low and high LSR in each condition during the fixed $\dot{H}$_prod_ walk, run and GXT, highlighting individual variability in sweating response between individuals.

**FIGURE 8:**
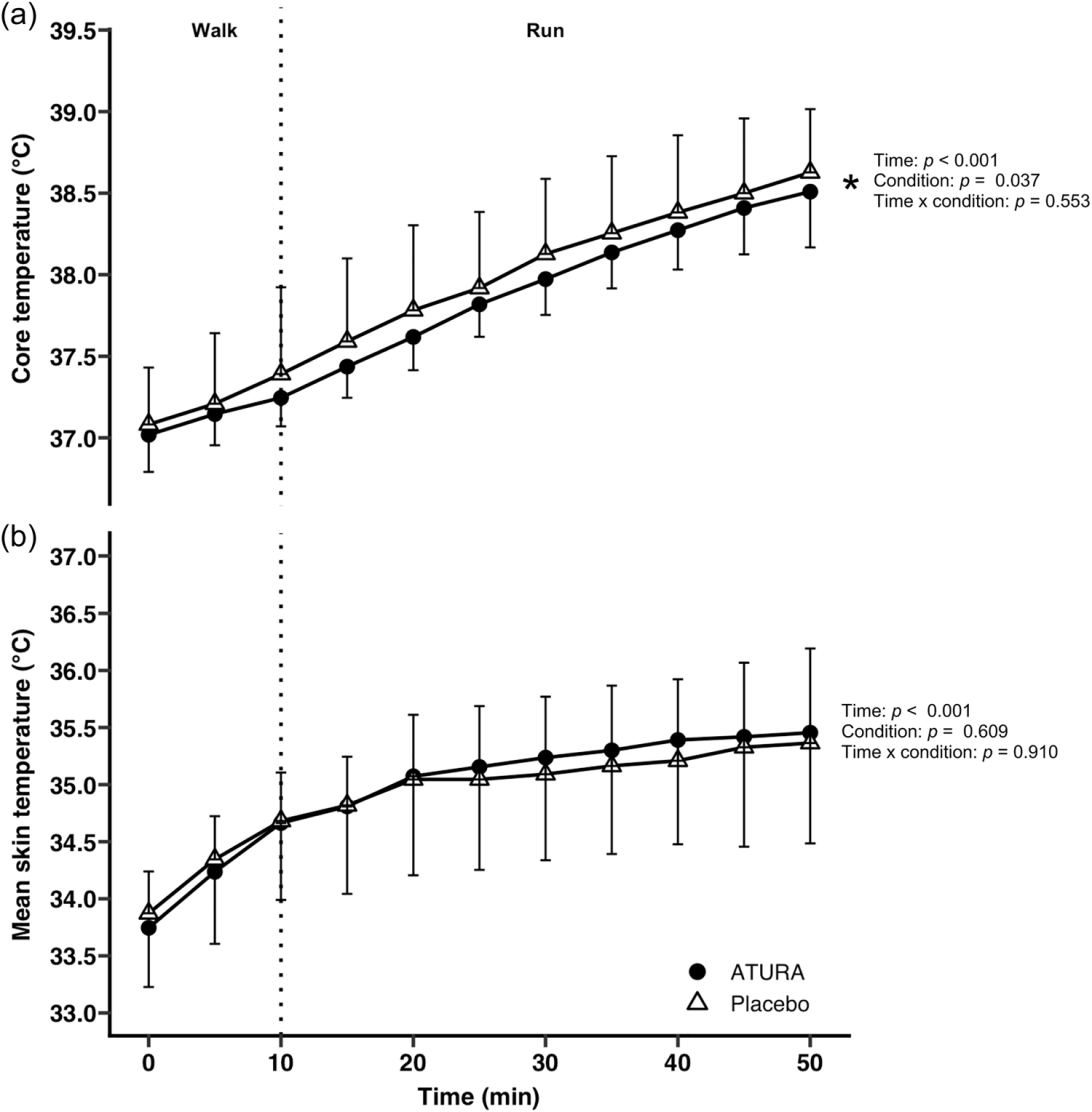
Core temperature (a) and skin temperature (b) following fixed heat production walk and run for ATURA or Placebo conditions. Vertical dotted lines represent the transitions between the walk and run phases. Core temperature (condition, *P* = 0.037; interaction, *P* = 0.553), skin temperature (condition, *P* = 0.609; interaction, *P* = 0.910). *Significant condition effect (*P* < 0.05; *n* = 12).

Both *T*_core_ and *T*_skin_ were analysed using a two‐way repeated‐measures ANOVA. During the trial, *T*_core_ increased with time across both conditions [*F*_(1,11)_ = 334.081, *P* < 0.001, η_p_^2^ = 0.972]. At the end of the walk, *T*_core_ was 37.2 ± 0.18 [37.1, 37.4]°C in the ATURA condition and 37.4 ± 0.53 [37.0, 37.7]°C in Placebo. At the end of the run, core temperature was 38.5 ± 0.34 [38.3, 38.7]°C in ATURA and 38.6 ± 0.39 [38.4, 38.9]°C in Placebo. There was a condition effect [*F*_(1,237)_ = 4.161, *P* = 0.037, η_p_^2^ = 0.131] but no interaction [*F*_(1,237)_ = 0.335, *P* = 0.553, η_p_^2^ = 0.080; Figure 8a]. *T*_skin_ increased [*F*_(1,11)_ = 67.614, *P* < 0.001, η_p_^2^ = 0.872] across the trial, with no condition effect [*F*_(1,237)_ = 0.263, *P* = 0.609, η_p_^2^ = 0.031] or interaction [*F*_(1,237)_ = 2.010, *P* = 0.910, η_p_^2^ = 0.130; Figure 8b]. At the end of the walk, *T*_skin_ was 34.7 ± 0.42 [34.4, 35.0]°C for Placebo and 34.7 ± 0.67 [34.2, 35.1]°C for ATURA; at the end of the run, *T*_skin_ was 35.4 ± 0.83 [34.8, 35.9]°C for Placebo and 35.5 ± 0.97 [34.8, 36.1]°C for ATURA. There were no trial order effects for *T*_core_ (*P* = 0.072) and *T*_skin_ (*P* = 0.158).

### 3.7 Perceptual responses

**FIGURE 9:**
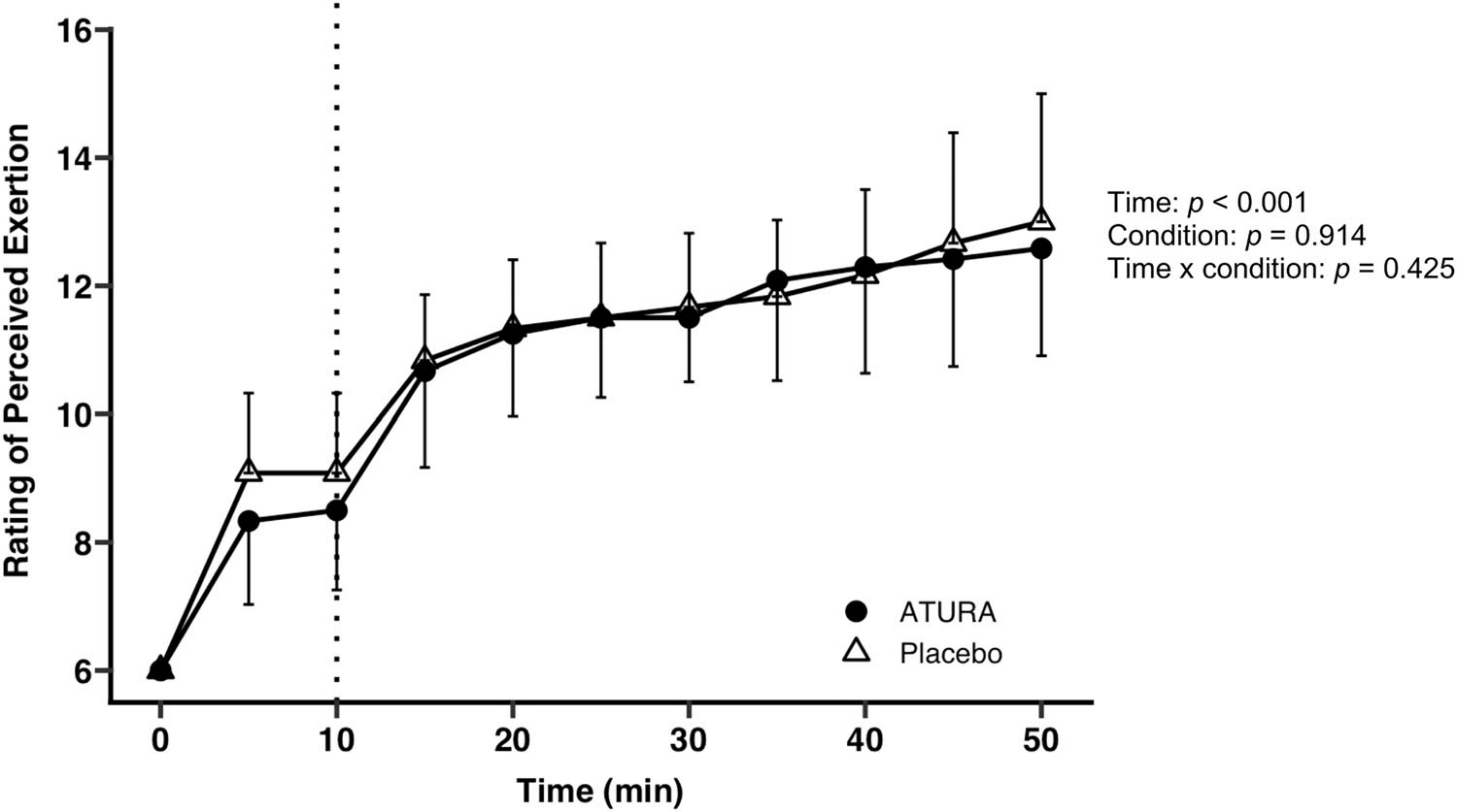
Rating of perceived exertion during the fixed heat production walk and run. Vertical dotted lines represent the transitions between the walk and run phases of the trial. Rating of perceived exertion (condition, *P* = 0.914; interaction, *P* = 0.425; *n* = 12).

During the trial, RPE increased with time across both conditions [*F*_(1,11)_ = 80.245, *P* < 0.001, η_p_^2^ = 0.042] and was similar between trials {Placebo vs. ATURA: 9.08 ± 1.24 [8.30, 9.87] vs. 8.5 ± 1.24 [7.71, 9.29] end‐walk; 13.0 ± 2.0 [11.7, 14.3] vs. 12.6 ± 1.68 [11.5, 13.6] beats/min end‐run}. There was no condition effect [*F*_(1,235)_ = 0.012, *P* = 0.914, η_p_^2^ = 0.020] or interaction [*F*_(1,235)_ = 0.638, *P* = 0.425, η_p_^2^ = 0.110; Figure 9]. Thermal sensation increased with time across both conditions [*F*_(11,229.01)_ = 21.680, *P* < 0.001, η_p_^2^ = 0.794; Table 3]. There was a condition effect [*F*_(1,229.01)_ = 1.331, *P* = 0.050, η_p_^2^ = 0.051], with no interaction [*F*_(10,229.01)_ = 1.784, *P* = 0.064, η_p_^2^ = 0.038]. Thermal comfort increased over time across both conditions [*F*_(11,229)_ = 19.120, *P* < 0.001, η_p_^2^ = 0.126; Table 3]. There was no condition effect for thermal comfort [*F*_(1,229)_ = 0.821, *P* = 0.371, η_p_^2^ = 0.201] or interaction [*F*_(10,229.01)_ = 0.630, *P* = 0.790, η_p_^2^ = 0.127].

**TABLE 3:** Thermal sensation and thermal comfort during the fixed heat production walk and run.

	0 min	10 min	20 min	30 min	40 min	50 min
Thermal sensation^*^						
ATURA	1 (1–1)	1 (1–2) [0.59, 1.24]	2 (2–2)	2 (2–2)	2 (2–3)	2 (2–3) [1.26, 2.32]
Placebo	1 (1–2)	2 (1–2) [0.36, 1.14]	2 (2–3)	3 (2–3)	3 (2–3)	3 (3–3) [1.66, 2.61]
Thermal comfort						
ATURA	1 (0–1)	1 (1–1) [0.59, 1.24]	1 (1–1)	1 (1–2)	2 (1–2)	2 (2–3) [1.26, 2.32]
Placebo	1 (0–1)	1 (0–1) [0.36, 1.14]	1 (0–2)	1 (1–2)	2 (1–2)	2 (2–3) [1.66, 2.61]

*Note*: Data are presented as the median and interquartile range (lower quartile–upper quartile range), with [95% confidence interval] at the end of walk (10 min) and run (50 min) (*n* = 12). Thermal sensation (condition, *P* = 0.050; interaction, *P* = 0.064), thermal comfort (condition, *P* = 0.371; interaction, *P* = 0.790).

* Significant condition effect (*P* < 0.05).

### 3.8 Partitional calorimetry and skin blood flow

**FIGURE 10:**
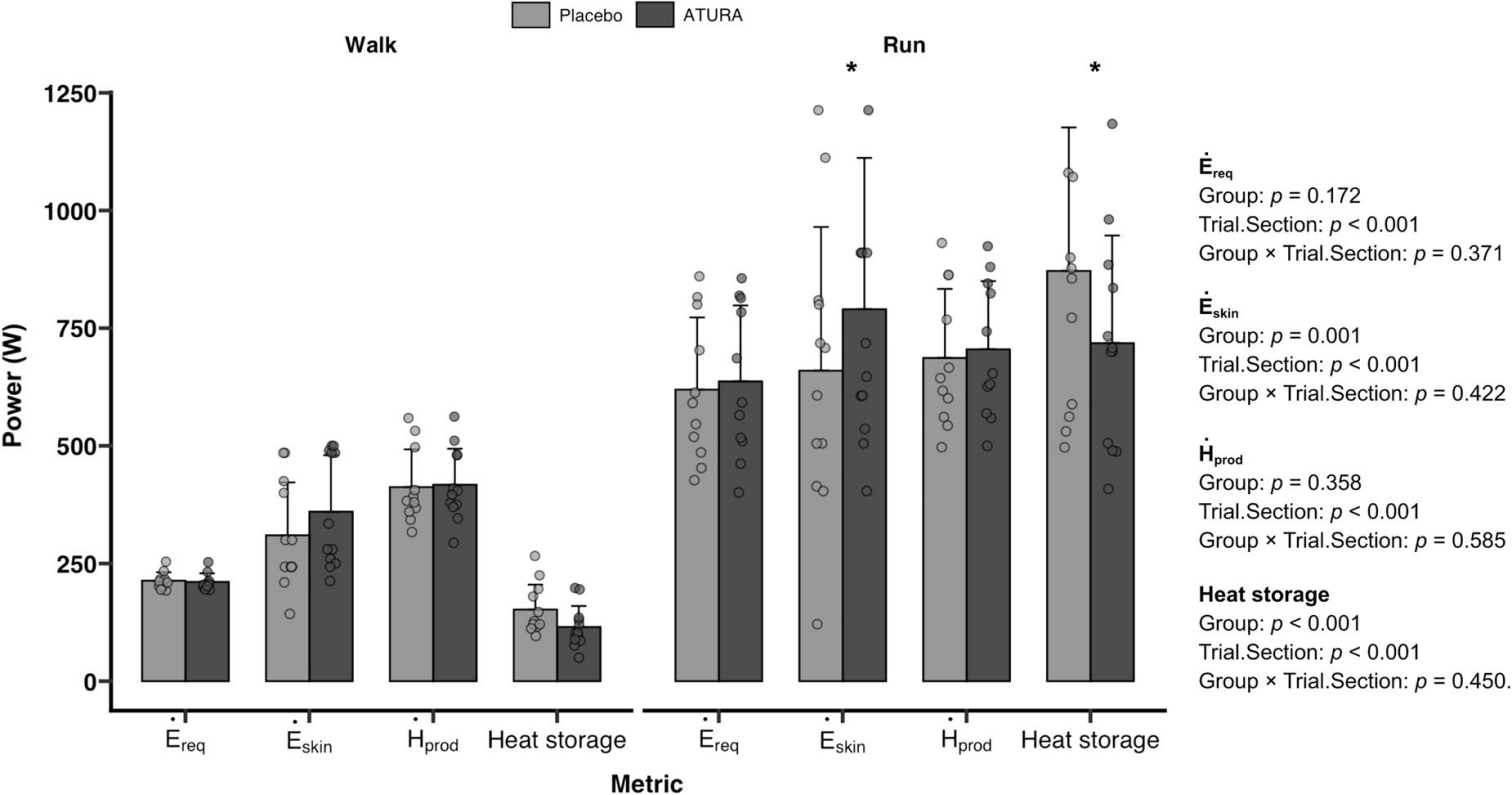
Mean evaporate requirement for heat balance (*Ė*_req_), evaporation at the skin surface (*Ė*_skin_), heat production ($\dot{H}$_prod_) and heat storage during the fixed heat production ($\dot{H}$_prod_) walk and run. *Ḣ*_prod_ (condition, *P* = 0.585; interaction, *P* = 0.585), *Ė*_req_ (condition, *P* = 0.371; interaction, *P* = 0.371), *Ė*_skin_ (condition, *P* = 0.001; interaction, *P* = 0.422), heat storage (condition, *P* = 0.030; interaction, *P* = 0.450). *Significant condition effect (*n* = 12).

Calculated SkBF {ATURA = 3.41 ± 0.62 L/min; Placebo = 3.33 ± 0.72 L/min; *P* = 0.781, 95% CI [−0.68, 0.52]} did not differ between conditions during the fixed $\dot{H}$_prod_ walk and run. There was a significant trial section (walk, run) and group interaction for all outcomes (*P* < 0.05). Heat production remained constant across each condition {walk: ATURA = 705 ± 145 W and Placebo = 687 ± 161 W; run: ATURA = 417 ± 76.4 W and Placebo = 412 ± 80 W [*F*_(1,20)_ = 0.308, *P* = 0.585, η_p_^2^ = 0.090]}. There was a condition effect for $\dot{E}$_skin_ [*F*_(1,23)_ = 20.520, *P* = 0.001, η_p_^2^ = 0.570] and heat storage [*F*_(1,23)_ = 30.3, *P* = 0.030, η_p_^2^ = 0.232] but no differences for $\dot{E}$_req_ [*F*_(1,23)_ = 0.841, *P* = 0.371, η_p_^2^ = 0.201] during the fixed $\dot{H}$_prod_ walk and run (Figure 10). For dry heat loss ($\dot{H}$_dry skin_), there were no differences between conditions {walk: ATURA = −6.38 ± 11.87 W and Placebo = −11.12 ± 9.64 W; run: ATURA = 13.06 ± 11.75 W, Placebo = 13.20 ± 9.14 W [*F*_(1,11)_ = 0.640, *P* = 0.444, η_p_^2^ = 0.070]}.

### 3.9 Vascular responses

No differences were observed between conditions for FMD {*t*_(11)_ = 0.142, *P* = 0.889, Cohen's *d* = 0.03, 95% CI [−0.739, 0.841]}, baseline diameter {*t*_(11)_ = 0.810, *P* = 0.431, Cohen's *d* = 0.02, 95% CI [−0.075, 0.035]}, maximum diameter {*t*_(11)_ = 0.775, *P* = 0.457, Cohen's *d* = 0.02, 95% CI [−0.080, 0.165]}, baseline shear rate {*t*_(11)_ = 0.501, *P* = 0.629, Cohen's *d* = 0.01, 95% CI [−16.63, 26.23]} and maximum shear rate {*t*_(11)_ = 0.10, *P* = 0.922, Cohen's *d* = 0.01, 95% CI [−106.58, 116.60]; Table 4}.

**TABLE 4:** Parameters of vascular endothelial function.

Variable	ATURA	Placebo
FMD (%)	7.3 ± 1.9	7.2 ± 2.7
Baseline diameter, mm	3.4 ± 0.5	3.4 ± 0.5
Peak diameter, mm	3.7 ± 0.6	3.6 ± 0.5
Baseline shear rate, s^−1^, AUC	191.8 ± 50.8	187.1 ± 27.9
Peak shear rate, s^−1^, AUC	708.8 ± 166.1	703.8 ± 174.2

*Note*: Data are presented as the mean ± SD (*n* = 12). FMD, *P* = 0.889; baseline diameter, *P* = 0.431; maximum diameter, *P* = 0.457; baseline shear rate, *P* = 0.629; maximum shear rate, *P* = 0.922.

Abbreviations: AUC, area under the curve; FMD, flow‐mediated dilatation.

## 4 DISCUSSION

In the present study, we investigated the effects of fava bean protein (ATURA) supplementation on exercise performance, sweating responses, *T*_core_, GIS and blood biomarkers of gut barrier integrity during a fixed‐$\dot{H}$_prod_ walk and run, followed by a GXT in a hot environment (35°C, 40% relative humidity). All measures of sweating were higher in the ATURA condition compared with Placebo, alongside a lower *T*_core_ and thermal sensation, despite no changes to ${\dot{V}}_{O_{2}\mathrm{peak}}$ or GXT performance. These thermoregulatory and perceptual effects were supported by lower circulating i‐FABP postexercise in the ATURA condition compared with placebo, with no adverse effects on self‐reported GIS. These findings demonstrate that ATURA supplementation improved thermoregulation during running in a hot environment through enhanced sweating, resulting in a lower *T*_core_, whilst ameliorating intestinal injury, without exacerbating GIS or changing a brief incremental test to exhaustion.

GIS were not different between conditions, indicating that the supplement did not exacerbate GIS relative to the placebo control. It has been reported that consumption of whey protein before and during exercise (∼15 g/20 min, ∼6% w/v) in a hot environment increased GIS in comparison to carbohydrate and water intake (Snipe et al., 2017). However, in the present study, ATURA was consumed 30 min before exercise, which might explain why GIS was unaffected. Therefore, the timing of protein ingestion pre‐exercise might be important for attenuating GIS. The assessment of individual AAs on exercise‐induced GIS is limited, but the available studies align with the present findings. For instance, l‐glutamine supplementation did not reduce GIS during a 60 min run (Pugh et al., 2017). Furthermore, Costa et al. (2023) investigated the effects of two proprietary AA beverage interventions, consumed twice daily for 7 days before and during a 2 h exertional heat stress running protocol (60% of maximal oxygen uptake, 35°C). These results suggest that AAs, either alone or in a blend, might not effectively alleviate exercise‐induced GIS. However, a limitation of the study by Costa et al. (2023) is that the GIS scale used to assess symptoms lacks specific validation for exercise contexts, which might impact the reliability of findings related to GIS during exercise. This limitation reflects a broader issue in exercise gastroenterology research, because there is currently no validated tool in the literature specifically designed for assessing exercise‐induced GIS.

Despite no significant reduction in GIS, we observed a 22% decrease in circulating i‐FABP levels postexercise in the ATURA condition compared with placebo. This finding supports previous research, demonstrating the protective effects of various protein sources, single AAs (e.g. l‐glutamine) and AA combinations on exercise‐induced intestinal damage during exercise in the heat (Pugh et al., 2017; Snipe et al., 2017; Costa et al., 2023). Specifically, Snipe et al. (2017) reported a 90% reduction in postexercise i‐FABP levels following whey protein hydrolysate intake (∼15 g/20 min, ∼6% w/v) compared with placebo. Key AAs in protein, such as l‐glutamine and l‐arginine, have been reported to enhance enterocyte proliferation, strengthen tight junctions and mitigate oxidative stress in intestinal epithelial cells (Beutheu et al., 2013; Costa et al., 2014; Zuhl et al., 2014). Moreover, Costa et al. (2023) observed a 50%–75% reduction in circulating i‐FABP using a specific blend of valine, aspartic acid, serine, threonine and tyrosine. Similar AA blends have also shown benefits in animal and in vitro models and might offer protection through improved cellular stability and enhanced epithelial homeostasis (Duan et al., 2014; Gupta et al., 2020; King et al., 2023; Yin et al., 2016). Although i‐FABP concentrations increased after exercise, this change did not reach statistical significance. It is possible that repeated carbohydrate feeding during the trial helped to maintain splanchnic perfusion (Snipe et al., 2017), thereby attenuating the rise in i‐FABP that might otherwise have occurred. Nevertheless, the present study demonstrates that a single, acute pre‐exercise dose of protein can provide protection against exercise‐induced intestinal damage. The evidence presented herein, including AA blends, whey protein and plant protein, suggests that various protein sources and AA combinations might offer some protective effects against exercise‐induced intestinal injury.

It is well established that exercise‐induced intestinal injury coincides with an acute increase in inflammatory markers, such as sCD14 and IL‐6 (Keirns et al., 2020). sCD14 serves as a co‐receptor for lipopolysaccharide, a marker of monocyte activation linked to endotoxin translocation during EIGS (Young et al., 2023). In the present study, no significant differences in sCD14 levels were observed between conditions. This contrasts with findings from the study by Costa et al. (2023), where reductions in these markers were reported with a proprietary AA blend, probably reflecting the greater thermal and GI strain of their 2 h running protocol compared with the present study. Although there are limited data on sCD14 responses to exercise, the present evidence suggests that responses are observed only with sufficiently intense or prolonged exercise stimuli (Costa et al., 2022). Likewise, IL‐6, a key cytokine in exercise‐induced inflammation, did not differ between groups postexercise, consistent with previous reports on whole protein (Snipe et al., 2017), which showed no attenuation of systemic inflammation during moderate exercise stress. However, consumption of the aforementioned proprietary AA blend during exercise in a hot environment reduced inflammatory markers, such as IL‐6 and IL‐8 (Costa et al., 2023), suggesting that AA dose and form might have an influence on systemic inflammatory responses related to gut‐derived endotoxaemia. These findings, alongside reductions in i‐FABP, suggest that ATURA might mainly target gut integrity rather than systemic inflammatory pathways, and further research with comprehensive inflammatory profiling is warranted.

A major finding was the thermoregulatory response to ATURA supplementation, characterized by increased WBSR and LSR of the back, resulting in a lower *T*_core_ compared with Placebo. Although the between‐condition difference in end‐exercise *T*_core_ was small, the effect was statistically significant and observed consistently across participants (83%), with values of 38.5 ± 0.34 [38.3, 38.7]°C in ATURA and 38.6 ± 0.39 [38.4, 38.9]°C in Placebo. Pre–post supplementation measurements of thermoregulatory variables and PV would clarify whether ATURA exerted acute pre‐exercise effects. Although the study was not powered to detect small effects in secondary variables, the consistent direction of response supports the possibility that this exploratory finding represents a true physiological effect and is worthy of further investigation. However, the between‐day error of rectal measures is −0.04°C, with 95% limits of agreement = −0.41°C to 0.33°C (Mee et al., 2015), which questions the clinical meaning of these differences. Although the direct mechanism for this finding was not determined here, this is supported by reports of increased sweat production following high‐volume consumption of other AAs, such as taurine (Page et al., 2019; Peel et al., 2024). However, this has not been the case for all studies, with other isolated AAs contained within the ATURA complex, such as l‐arginine (an NO precursor), reported by a recent meta‐analysis to have no significant effect on sweating and *T*_core_ responses to heat exposure (Peel et al., 2025). Moreover, individual supplementation with l‐arginine (10 g) or l‐citrulline (6 g/day; an l‐arginine precursor) had no effect on thermoregulatory responses during exercise in the heat (Cable et al., 2024; Tyler et al., 2016). However, plasma NO concentration was not measured in either study, and it remains unclear whether any increase in plasma l‐arginine or l‐citrulline translated to higher NO production. These findings suggest that the enhanced sweating response observed with ATURA is unlikely to be attributable to the 4.6 g/day of arginine via its effect on the NOS pathway, despite the established role of this pathway on eccrine sweating (Mack, 2020). Alternatively, it is plausible that transient shifts in osmotic load imparted by the high plasma AA availability, particularly AAs such as l‐glutamine and l‐proline (Dall'Asta et al., 1999), permitted greater PV expansion in the ATURA condition. However, in the present study there was no clear difference in PV change between conditions, and these data do not provide empirical support for this mechanism. The method used to estimate PV (pre‐ and postexercise capillary sampling) is relatively insensitive to short‐lived or subtle fluctuations, hence any such effects might not have been detectable with the present approach. Furthermore, given the capacity for interstitial fluid compartments transiently to increase both fluid and AA composition during exercise‐related stress (Zhang et al., 2019), the availability of small, neutral AAs (such as alanine, glycine and serine contained in ATURA) or those with osmotic roles reported previously (i.e. taurine; Cuisinier et al., 2002; Peel et al., 2024) could have played a role in transiently shifting interstitial fluid osmotic pressure, thus facilitating fluid replenishment for thermal sweating to occur. It is also possible that the 8‐day ATURA supplementation period was sufficient to increase albumin synthesis and subsequent plasma concentrations, as was reported after 5 days of a mixed protein supplement with a similar AA profile to ATURA, which increased PV and enhanced thermoregulatory responses (Goto et al., 2010; Okazaki et al., 2009). Collectively, these proposed mechanisms should be interpreted as plausible explanations that require targeted, mechanistic investigation in future studies to establish the potential effects of ATURA on both plasma and interstitial fluid balance dynamics during exercise.

The corresponding increase in evaporative cooling (*Ė*_skin_) in the ATURA condition, alongside a reduction in heat storage, highlights a more effective thermoregulatory response compared with placebo. These findings are particularly notable given that $\dot{H}$_prod_, HR, oxygen consumption and RER were consistent across trials, indicating that the enhanced heat dissipation was not attributable to differences in exercise intensity, cardiovascular strain or metabolic workload. Given that most metabolic heat during exercise must be dissipated to maintain thermal equilibrium (Kenny & McGinn, 2017), the observed increase in *Ė*_skin_ with ATURA directly reflects the greater availability of sweat at the surface of the skin.

Another plausible mechanism for the enhanced thermoregulatory response in the ATURA condition is the elevation in serum HSP70, given that postexercise HSP70 was significantly higher following ATURA supplementation compared with placebo. HSPs play a crucial role in cellular protection during thermal stress and can enhance heat tolerance during exercise (Krüger et al., 2019). Although promising, the effect of AA or whole protein supplementation on HSPs and subsequent thermoregulatory responses is not well established. It has been reported that l‐arginine and l‐glutamine can increase HSP70 expression in both muscle and peripheral blood mononuclear cells (Hamiel et al., 2009; Moura et al., 2017; Zuhl et al., 2015). l‐Glutamine, which is metabolically linked to glutamate (the form provided in the present study, 8.28 g/day), might also contribute to this protective effect by activating HSPs (e.g. via HSF‐1) and inhibiting pro‐inflammatory pathways, such as nuclear factor‐κB (Morrison et al., 2006; Singleton & Wischmeyer, 2006). However, given that only serum HSP70 was measured here, this might not directly reflect intracellular HSP/HSF‐1 signalling. Animal models have demonstrated that whey protein can increase tissue‐specific HSP70 responses following physical exertion (De Moura et al., 2013), suggesting a broader role for dietary proteins in modulating HSP responsiveness. Plausibly, the concurrent rise in HSP70 and reduction in the intestinal injury marker i‐FABP suggest a potential protective effect on the gut environment. Mechanistic evidence indicates that HSP70 stabilizes tight junction proteins, thereby preserving barrier integrity and limiting endotoxin translocation and systemic inflammation (Dokladny et al. 2006; Zuhl et al. 2015). Although this pathway offers a plausible explanation for the observed decrease in *T*_core_, these relationships remain speculative and require direct mechanistic verification. Collectively, these findings support the role of HSP70 as a biomarker of heat stress resilience, warranting further investigation into the precise mechanisms for its response to protein ingestion.

Exercise tolerance did not change with ATURA supplementation, given that there were no effects on ${\dot{V}}_{O_{2}\mathrm{peak}}$ or GXT time in the heat or during the baseline incremental test. It was considered that the thermoregulatory effects of the supplement would translate to extended endurance time to exhaustion in the heat. However, the small magnitude of the *T*_core_ difference {ATURA: 38.5 ± 0.34 [38.3, 38.7]°C; Placebo: 38.6 ± 0.39 [38.4, 38.9]°C}, alongside unchanged HR, ${\dot{V}}_{O_{2}}$ and RPE, suggests that these changes were unlikely to be physiologically meaningful for exercise tolerance in this protocol. A trial‐order effect was detected for ${\dot{V}}_{O_{2}\mathrm{peak}}$ in the heat (*P *= 0.020), with significant increase at the second visit, independent of supplementation and without corresponding changes in workload or performance. The present data concur with studies conducted in thermoneutral environments, where no improvements in ${\dot{V}}_{O_{2}\mathrm{peak}}$ and endurance exercise tolerance are also commonly reported (Forbes & Bell, 2019; Hansen et al., 2016; Schroer et al., 2014). Studies investigating pre‐ or per‐exercise protein supplementation on exercise tolerance are notably scarce, particularly in hot environments. Co‐ingestion of protein with carbohydrate has been investigated more frequently, given the potential for enhanced glucose availability and performance benefits, but mixed findings have been reported (Nielsen et al., 2020; Zhao et al., 2024). The role of specific AAs, rather than whole protein sources, during exercise in hot environments has been explored (Kephart et al., 2016; Mittleman et al., 1998; Pugh et al., 2017; Tumilty et al., 2011), with both tyrosine (∼14.8%; Tumilty et al., 2011) and BCAAs (∼11.1%; Mittleman et al. 1998) enhancing exercise performance through 1 h time to exhaustion (TTE) and GXT (∼18 min), respectively, but without any thermoregulatory changes. Although large, isolated doses of AAs, such as BCAAs (∼6 g/day) or tyrosine (∼150 mg/kg body mass), appear to confer ergogenic effects, potentially via centrally mediated mechanisms (Newsholme & Blomstrand, 2006; Williams, 2005), these effects were not observed after ATURA supplementation and might be a consequence of the lower AA doses. Given the distinct physiological roles of AAs, it is possible that the benefits of protein supplementation might be leveraged more optimally by staggering the supplementation of specific AAs across the pre‐ to postexercise period, but further research is required to confirm this.

For perceptual responses during the trial, thermal sensation was lower in the ATURA condition compared with Placebo, whereas RPE and thermal comfort were similar between groups. Supplementation with single AAs, including glutamine (Pugh et al., 2017), tyrosine (Tumilty et al., 2011) and taurine (Peel et al., 2024), AA blends (Costa et al., 2023) and BCAAs (Mittleman et al., 1998), during exercise in thermally stressful environments showed no changes in thermal comfort (Pugh et al., 2017; Costa et al., 2023; Watson et al., 2004; Peel et al., 2024) or thermal sensation (Mittleman et al., 1998; Tumilty et al., 2011; Peel et al., 2024). These findings support the present results for thermal comfort but not thermal sensation. The lower thermal sensation in the ATURA condition suggests that participants felt less hot during the trial but not necessarily more comfortable. Although variation in skin temperature is associated with thermal sensation (Flouris & Schlader., 2015), no differences were observed between conditions, hence other drivers, such as lower *T*_core_ and elevated WBSR, might have alleviated the perceived heat strain.

There were no significant differences observed between conditions for vascular function, as determined by FMD percentage, baseline or maximum diameter, or shear rate of the brachial artery. Amino acids, such as l‐arginine and l‐citrulline, are established vasodilators, owing to their role in NO production, with l‐citrulline reported to improve endothelial function (Maharaj et al., 2022; Morita et al., 2013). Furthermore, an acute dose of whey protein supplementation has been reported to improve the FMD response in overweight adults (Ballard et al., 2009) and physically fit adults (de Oliveira et al., 2020), which is inconsistent with the results of the present study. This discrepancy might reflect differences in AA composition; Da Silva et al. (2017) demonstrated that greater BCAA concentration of whey protein increased endothelial NO synthase expression and improved endothelium‐dependent dilatation in vitro. Specifically, ATURA contains 137.5 mg/g BCAAs, which is ∼2.6‐fold lower than the 352.1 mg/g found in a whey protein source (de Oliveira et al., 2020). The lower BCAA content might result in less stimulation of endothelial NO synthase and, consequently, a reduced effect on NO‐mediated vascular function; however, a whole protein complex of other AA combinations might be more beneficial for thermoregulation through enhanced sweating, as evidenced in the present findings. However, these responses are likely to be mediated by NO only in part, with reactive hyperaemia involving additional pathways, such as prostanoids, BKCa channels and cholinergic K^+^ channel signalling, independent of NO (Fujii et al., 2016; Lorenzo & Minson, 2007). Additionally, the cohort in the present study consisted of trained individuals, and responses might have been blunted as observed by Green et al. (2013), who reported that chronic training‐induced arterial remodelling in athletes can mask further improvements in FMD, resulting in a blunted or unchanged FMD response despite superior vascular health. Therefore, differences in protein composition and training status are likely to have contributed to the conflicting findings of the present study.

### 4.1 Limitations

This study provides new insights into the effects of plant‐based protein ingestion on thermoregulation, intestinal damage and GIS; however, there are some limitations to be acknowledged. First, despite its reported reliability (Gaskell et al., 2019), the GIS scale used herein has not yet undergone formal validation in athletes, which means that the reporting of GI symptoms might not be captured accurately. Second, the exercise trial duration might have been insufficient to elicit the greater GI disturbances reported in prolonged endurance events (Gaskell et al., 2023). Third, carbohydrate‐containing beverages were provided as part of the trial to exacerbate GIS. However, carbohydrate ingestion during exercise has also been reported to attenuate increases in intestinal permeability and enterocyte injury markers (Snipe et al., 2017). Therefore, this might have blunted any changes in intestinal injury detected in the present study. Fourth, serum HSP70 concentrations might primarily reflect a generalized systemic stress response (Lechner et al., 2018; Qu et al., 2015) and can indicate beneficial cellular stress responses relevant to gut health (Sepponen & Pösö, 2006; Wang et al., 2018). Serum and cellular HSP70 represent related but distinct compartments (Hunter‐Lavin et al., 2004; Yamada et al., 2007), but to enhance the findings of the present study, tissue‐specific assessment of cellular protective mechanisms and molecular changes within the gut epithelium will be essential in future studies of this supplement. Fifth, self‐reported calendar counting prevented confirmation of the menstrual phase or ovarian hormone levels, potentially introducing residual variability (Elliott‐Sale et al., 2025). Sixth, skin blood flow was estimated rather than being measured directly; running motion artefact would have compromised Doppler reliability. Lastly, the absence of plasma and interstitial fluid AA profiling precludes confirmation of supplement bioavailability and absorption at exercise time points and highlights an essential priority for future mechanistic studies.

## 5 CONCLUSION

This study is the first to demonstrate that fava bean protein (ATURA) supplementation can positively influence intestinal epithelial integrity during exercise in hot conditions, as indicated by reduced circulating i‐FABP. This finding suggests a potential protective effect on the gut barrier. ATURA supplementation did not alter GIS, indicating that pre‐exercise consumption was well tolerated in this exercise setting. Secondary and exploratory analyses suggested that ATURA might influence thermoregulatory responses, evidenced by higher WBSR and back LSR, lower *T*_core_ and elevated postexercise circulating HSP70. Although the ∼0.1°C difference in *T*_core_ falls within the measurement error (Mee et al., 2015) and requires confirmation in larger samples, the concurrent increases in WBSR and back LSR and reduced heat storage are consistent with enhanced heat dissipation and support the plausibility of a thermoregulatory effect. Although these trends provide preliminary insights into possible mechanisms supporting thermal tolerance, they should be interpreted cautiously owing to their exploratory nature. Collectively, these findings support the potential of fava bean protein as a pre‐exercise supplement that might help to maintain gut integrity and thermoregulatory function during running in the heat, warranting replication and further mechanistic investigation.

## AUTHOR CONTRIBUTIONS

Robyn Aitkenhead, Shane M. Heffernan and Mark Waldron designed the initial concept, and Robyn Aitkenhead, Shane M. Heffernan and Mark Waldron refined the study design. Robyn Aitkenhead conducted the experimental procedures with specific technical expertise from Shane M. Heffernan and Mark Waldron Robyn Aitkenhead drafted the initial manuscript, data analysis and presentation, and all authors were involved in subsequent drafting and approval of the final manuscript. All authors agree to be accountable for all aspects of the work in ensuring that questions related to the accuracy or integrity of any part of the work are appropriately investigated and resolved. All persons designated as authors qualify for authorship, and all those who qualify for authorship are listed.

## CONFLICT OF INTEREST

R.A., S.M.H. and M.W. have received funding from Marigot Ltd. The authors have no further conflicts to declare.

## Supporting information



Supporting Information

## Data Availability

Data can be made available upon reasonable request.

## References

[eph70271-bib-0001] American College of Sports Medicine . (2014). L. S. Pescatello , R. Arena , D. Riebe , & P. D. Thompson (Eds.), ACSM's guidelines for exercise testing and prescription (9th ed.). Wolters Kluwer/Lippincott Williams & Wilkins. 10.1249/JSR.0b013e31829a68cf 23851406

[eph70271-bib-0002] Badjona, A. , Bradshaw, R. , Millman, C. , Howarth, M. , & Dubey, B. (2023). Faba bean processing: Thermal and non‐thermal processing on chemical, antinutritional factors, and pharmacological properties. Molecules, 28(14), 5431. 37513301 10.3390/molecules28145431 PMC10383711

[eph70271-bib-0003] Bai, Y. , Sun, L. , Yang, T. , Sun, K. , Chen, J. , & Hui, R. (2009). Increase in fasting vascular endothelial function after short‐term oral L‐arginine is effective when baseline flow‐mediated dilation is low: A meta‐analysis of randomized controlled trials. The American Journal of Clinical Nutrition, 89(1), 77–84. 19056561 10.3945/ajcn.2008.26544

[eph70271-bib-0121] Ballard, K. D. , Kupchak, B. R. , Volk, B. M. , Mah, E. , Shkreta, A. , Liptak, C. , Ptolemy, A. S. , Kellogg, M. S. , Bruno, R. S. , Seip, R. L. , Maresh, C. M. , Kraemer, W. J. , & Volek, J. S. (2013). Acute effects of ingestion of a novel whey‐derived extract on vascular endothelial function in overweight, middle‐aged men and women. The British Journal of Nutrition, 109(5), 882–893. 22691263 10.1017/S0007114512002061

[eph70271-bib-0005] Ballard, K. D. , Bruno, R. S. , Seip, R. L. , Quann, E. E. , Volk, B. M. , Freidenreich, D. J. , Kawiecki, D. M. , Kupchak, B. R. , Chung, M. Y. , Kraemer, W. J. , & Volek, J. S. (2009). Acute ingestion of a novel whey‐derived peptide improves vascular endothelial responses in healthy individuals: A randomized, placebo controlled trial. Nutrition Journal, 8, 1–11. 19624856 10.1186/1475-2891-8-34 PMC2723133

[eph70271-bib-0007] Basson, A. R. , Gomez‐Nguyen, A. , LaSalla, A. , Buttó, L. , Kulpins, D. , Warner, A. , Di Martino, L. , Ponzani, G. , Osme, A. , Rodriguez‐Palacios, A. , & Cominelli, F. (2021). Replacing animal protein with soy‐pea protein in an “American diet” controls murine crohn disease–like ileitis regardless of firmicutes: Bacteroidetes ratio. The Journal of Nutrition, 151(3), 579–590. 33484150 10.1093/jn/nxaa386 PMC7948210

[eph70271-bib-0122] Bedford, T. (1936). The warmth factor in comfort at work: A physiological study of heating and ventilation (Report No. 76). Medical Research Council, Industrial Health Research Board. H.M. Stationery Office.

[eph70271-bib-0010] Beutheu, S. , Ghouzali, I. , Galas, L. , Déchelotte, P. , & Coëffier, M. (2013). Glutamine and arginine improve permeability and tight junction protein expression in methotrexate‐treated Caco‐2 cells. Clinical Nutrition, 32(5), 863–869. 23428392 10.1016/j.clnu.2013.01.014

[eph70271-bib-0012] Borg, G. A. V. (1982). Psychophysical bases of perceived exertion. Medicine & Science in Sports & Exercise, 14(5), 377–381. 7154893

[eph70271-bib-0013] Bovenschen, H. J. , Janssen, M. J. R. , Van Oijen, M. G. H. , Laheij, R. J. F. , Van Rossum, L. G. M. , & Jansen, J. B. M. J. (2006). Evaluation of a gastrointestinal symptoms questionnaire. Digestive Diseases and Sciences, 51(9), 1509–1515. 16927133 10.1007/s10620-006-9120-6

[eph70271-bib-0014] Brennan, J. L. , Keerati‐U‐Rai, M. , Yin, H. , Daoust, J. , Nonnotte, E. , Quinquis, L. , St‐Denis, T. , & Bolster, D. R. (2019). Differential responses of blood essential amino acid levels following ingestion of high‐quality plant‐based protein blends compared to whey protein—A double‐blind randomized, cross‐over, clinical trial. Nutrients, 11(12), 2987. 31817691 10.3390/nu11122987 PMC6950667

[eph70271-bib-0015] Bruce, R. , Kusumi, F. , & Hosmer, D. (1973). Maximal oxygen intake and nomographic assessment of functional aerobic impairment in cardiovascular disease. American heart journal, 85(4), 546–562. 4632004 10.1016/0002-8703(73)90502-4

[eph70271-bib-0016] Cable, T. G. , Funnell, M. P. , Reynolds, K. M. , Hudson, E. F. , Macrae, H. Z. , Johnson, D. A. , Taylor, L. , Heaney, L. M. , Mears, S. A. , Bailey, S. J. , & James, L. J. (2024). 7 days of L‐citrulline supplementation does not improve running performance in the heat whilst in a hypohydrated state. European Journal of Applied Physiology, 125(5), 1411–1421. 39699639 10.1007/s00421-024-05671-4 PMC12055621

[eph70271-bib-0017] Cathcart, A. J. , Murgatroyd, S. R. , McNab, A. , Whyte, L. J. , & Easton, C. (2011). Combined carbohydrate–protein supplementation improves competitive endurance exercise performance in the heat. European Journal of Applied Physiology, 111, 2051–2061. 21259024 10.1007/s00421-011-1831-5

[eph70271-bib-0020] Chinevere, T. D. , Sawyer, R. D. , Creer, A. R. , Conlee, R. K. , & Parcell, A. C. (2002). Effects of L‐tyrosine and carbohydrate ingestion on endurance exercise performance. Journal of applied physiology, 93(5), 1590–1597. 12381742 10.1152/japplphysiol.00625.2001

[eph70271-bib-0124] Cohen, J. (1988). Statistical power analysis for the behavioral sciences (2nd ed.). Lawrence Erlbaum Associates.

[eph70271-bib-0021] Costa, K. A. , Soares, A. D. N. , Wanner, S. P. , dos Santos, R. D. G. C. , Fernandes, S. O. A. , dos Santos Martins, F. , Nicoli, J. R. , Coimbra, C. C. , & Cardoso, V. N. (2014). L‐arginine supplementation prevents increases in intestinal permeability and bacterial translocation in male Swiss mice subjected to physical exercise under environmental heat stress. The Journal of nutrition, 144(2), 218–223. 24259555 10.3945/jn.113.183186

[eph70271-bib-0022] Costa, R. J. , Miall, A. , Khoo, A. , Rauch, C. , Snipe, R. , Camões‐Costa, V. , & Gibson, P. (2017). Gut‐training: The impact of two weeks repetitive gut‐challenge during exercise on gastrointestinal status, glucose availability, fuel kinetics, and running performance. Applied Physiology, Nutrition, and Metabolism, 42(5), 547–557. 10.1139/apnm-2016-0453 28177715

[eph70271-bib-0023] Costa, R. J. , Gaskell, S. K. , McCubbin, A. J. , & Snipe, R. M. (2020). Exertional‐heat stress‐associated gastrointestinal perturbations during Olympic sports: Management strategies for athletes preparing and competing in the 2020 Tokyo Olympic Games. Temperature, 7(1), 58–88. 10.1080/23328940.2019.1597676 PMC7053925 32166105

[eph70271-bib-0024] Costa, R. J. , Henningsen, K. , Gaskell, S. K. , Alcock, R. , Mika, A. , Rauch, C. , Cheuvront, S. N. , Blazy, P. , & Kenefick, R. (2023). Amino acid‐based beverage interventions ameliorate exercise‐induced gastrointestinal syndrome in response to exertional‐heat stress: The heat exertion amino acid technology (HEAAT) study. International Journal of Sport Nutrition and Exercise Metabolism, 33(4), 230–242. 37225167 10.1123/ijsnem.2023-0025

[eph70271-bib-0025] Costa, R. J. S. , Young, P. , Gill, S. K. , Snipe, R. M. J. , Gaskell, S. , Russo, I. , & Burke, L. M. (2022). Assessment of Exercise‐Associated Gastrointestinal Perturbations in Research and Practical Settings: Methodological Concerns and Recommendations for Best Practice. International Journal of Sport Nutrition and Exercise Metabolism, 32(8), 387–418. 35963615 10.1123/ijsnem.2022-0048

[eph70271-bib-0026] Cramer, M. N. , & Jay, O. (2019). Partitional calorimetry. Journal of Applied Physiology, 126(2), 267–277. 30496710 10.1152/japplphysiol.00191.2018 PMC6397408

[eph70271-bib-0027] Cuisinier, C. , Michotte de Welle, J. , Verbeeck, R. K. , Poortmans, J. R. , Ward, R. , Sturbois, X. , & Francaux, M. (2002). Role of taurine in osmoregulation during endurance exercise. European Journal of Applied Physiology, 87, 489–495. 12355187 10.1007/s00421-002-0679-0

[eph70271-bib-0028] Da Silva, M. S. , Bigo, C. , Barbier, O. , & Rudkowska, I. (2017). Whey protein hydrolysate and branched‐chain amino acids downregulate inflammation‐related genes in vascular endothelial cells. Nutrition Research, 38, 43–51. 28381353 10.1016/j.nutres.2017.01.005

[eph70271-bib-0029] Dall'Asta, V. , Bussolati, O. , Sala, R. , Parolari, A. , Alamanni, F. , Biglioli, P. , & Gazzola, G. C. (1999). Amino acids are compatible osmolytes for volume recovery after hypertonic shrinkage in vascular endothelial cells. American Journal of Physiology‐Cell Physiology, 276(4), C865–C872. 10.1152/ajpcell.1999.276.4.C865 10199817

[eph70271-bib-0030] De Moura, C. S. , Lollo, P. C. B. , Morato, P. N. , Carneiro, E. M. , & Amaya‐Farfan, J. (2013). Whey protein hydrolysate enhances the exercise‐induced heat shock protein (HSP70) response in rats. Food Chemistry, 136(3–4), 1350–1357. 23194534 10.1016/j.foodchem.2012.09.070

[eph70271-bib-0031] Dill, D. B. , & Costill, D. L. (1974). Calculation of percentage changes in volumes of blood, plasma, and red cells in dehydration. Journal of Applied Physiology, 37(2), 247–248. 4850854 10.1152/jappl.1974.37.2.247

[eph70271-bib-0032] Dokladny, K. , Moseley, P. L. , & Ma, T. Y. (2006). Physiologically relevant increase in temperature causes an increase in intestinal epithelial tight junction permeability. American Journal of Physiology‐Gastrointestinal and Liver Physiology, 290(2), G204–G212. 16407590 10.1152/ajpgi.00401.2005

[eph70271-bib-0033] Du Bois, D. , & Du Bois, E. (1916). Clinical calorimetry: Tenth paper a formula to estimate the approximate surface area if height and weight be known. Archives of Internal Medicine, 17, 863–871.

[eph70271-bib-0034] de Oliveira, E. P. , Burini, R. C. , & Jeukendrup, A. (2014). Gastrointestinal complaints during exercise: Prevalence, etiology, and nutritional recommendations. Sports Medicine, 44, 79–85. 10.1007/s40279-014-0153-2 PMC4008808 24791919

[eph70271-bib-0035] Duan, J. , Yin, J. , Wu, M. , Liao, P. , Deng, D. , Liu, G. , Wen, Q. , Wang, Y. , Qiu, W. , Liu, Y. , Wu, X. , Ren, W. , Tan, B. , Chen, M. , Xiao, H. , Wu, L. , Li, T. , Nyachoti, C. M. , Adeola, O. , & Yin, Y. (2014). Dietary glutamate supplementation ameliorates mycotoxin‐induced abnormalities in the intestinal structure and expression of amino acid transporters in young pigs. PLoS ONE, 9(11), e112357. 25405987 10.1371/journal.pone.0112357 PMC4236086

[eph70271-bib-0036] Elite Sports Nutrition . (2024). Designer flavour powder. Elite Sports Nutrition. https://uk.esn.com/products/designer‐flavor‐powder?variant=47947636638011

[eph70271-bib-0037] Ellis, A. C. , Patterson, M. , Dudenbostel, T. , Calhoun, D. , & Gower, B. (2016). Effects of 6‐month supplementation with β‐hydroxy‐β‐methylbutyrate, glutamine and arginine on vascular endothelial function of older adults. European journal of clinical nutrition, 70(2), 269–273. 26306566 10.1038/ejcn.2015.137 PMC4740211

[eph70271-bib-0038] Elliott‐Sale, K. J. , Altini, M. , Doyle‐Baker, P. , Ferrer, E. , Flood, T. R. , Harris, R. , Impellizzeri, F. M. , de Jonge, X. J. , Kryger, K. O. , Lewin, G. , Lebrun, C. M. , McCall, A. , Nimphius, S. , Phillips, S. M. , Swinton, P. A. , Taylor, M. , Verhagen, E. , & Burden, R. J. (2025). Why we must stop assuming and estimating menstrual cycle phases in laboratory and field‐based sport related research. Sports Medicine, 55(6), 1339. 40085421 10.1007/s40279-025-02189-3 PMC12152053

[eph70271-bib-0039] Flouris, A. D. , & Schlader, Z. (2015). Human behavioral thermoregulation during exercise in the heat. Scandinavian Journal of Medicine & Science in Sports, 25, 52–64. 25943656 10.1111/sms.12349

[eph70271-bib-0040] Forbes, S. C. , & Bell, G. J. (2019). Whey protein isolate supplementation while endurance training does not Alter cycling performance or immune responses at rest or after exercise. Frontiers in Nutrition, 6, 19. 30881958 10.3389/fnut.2019.00019 PMC6406070

[eph70271-bib-0041] Fujii, N. , Notley, S. R. , Minson, C. T. , & Kenny, G. P. (2016). Administration of prostacyclin modulates cutaneous blood flow but not sweating in young and older males: roles for nitric oxide and calcium‐activated potassium channels. The Journal of Physiology, 594(21), 6419–6429. 27511105 10.1113/JP273174 PMC5088240

[eph70271-bib-0128] Gaskell, S. K. , Burgell, R. , Wiklendt, L. , Dinning, P. G. , & Costa, R. J. S. (2023). Impact of exercise duration on gastrointestinal function and symptoms. Journal of Applied Physiology (Bethesda, Md. : 1985), 134(1), 160–171. 36476157 10.1152/japplphysiol.00393.2022

[eph70271-bib-0042] Gaskell, S. K. , Rauch, C. E. , & Costa, R. J. S. (2021). Gastrointestinal assessment and therapeutic intervention for the management of exercise‐associated gastrointestinal symptoms: A case series translational and professional practice approach. Frontiers in Physiology, 12, 719142. 34557109 10.3389/fphys.2021.719142 PMC8452991

[eph70271-bib-0123] Gaskell, S. K. , Snipe, R. M. J. , & Costa, R. J. S. (2019). Testretest reliability of a modified visual analog scale assessment tool for determining incidence and severity of gastrointestinal symptoms in response to exercise stress. International Journal of Sport Nutrition and Exercise Metabolism, 29(4), 411–419. 30632417 10.1123/ijsnem.2018-0215

[eph70271-bib-0043] Gil, M. , Rudy, M. , Duma‐Kocan, P. , Stanisławczyk, R. , Krajewska, A. , Dziki, D. , & Hassoon, W. H. (2024). Sustainability of alternatives to animal protein sources, a comprehensive review. Sustainability, 16(17), 7701.

[eph70271-bib-0044] Green, D. J. , Rowley, N. , Spence, A. , Carter, H. , Whyte, G. , George, K. , Naylor, L. H. , Cable, N. T. , Dawson, E. A. , & Thijssen, D. H. J. (2013). Why isn't flow‐mediated dilation enhanced in athletes? Medicine & Science in Sports & Exercise, 45(1), 75–82. 22843111 10.1249/MSS.0b013e318269affe

[eph70271-bib-0045] Gorissen, S. H. , Crombag, J. J. , Senden, J. M. , Waterval, W. H. , Bierau, J. , Verdijk, L. B. , & van Loon, L. J. (2018). Protein content and amino acid composition of commercially available plant‐based protein isolates. Amino Acids, 50, 1685–1695. 30167963 10.1007/s00726-018-2640-5 PMC6245118

[eph70271-bib-0046] Goto, M. , Okazaki, K. , Kamijo, Y. I. , Ikegawa, S. , Masuki, S. , Miyagawa, K. , & Nose, H. (2010). Protein and carbohydrate supplementation during 5‐day aerobic training enhanced plasma volume expansion and thermoregulatory adaptation in young men. Journal of Applied Physiology, 109(4), 1247–1255. 20689095 10.1152/japplphysiol.00577.2010

[eph70271-bib-0047] Gullón, P. , Gullón, B. , Tavaria, F. , Vasconcelos, M. , & Gomes, A. M. (2015). In vitro fermentation of lupin seeds (*Lupinus albus*) and broad beans (*Vicia faba*): Dynamic modulation of the intestinal microbiota and metabolomic output. Food & Function, 6(10), 3316–3322. 26252418 10.1039/c5fo00675a

[eph70271-bib-0048] Gupta, R. , Yin, L. , Grosche, A. , Lin, S. , Xu, X. , Guo, J. , Vaught, L. A. , Okunieff, P. G. , & Vidyasagar, S. (2020). An amino acid–Based oral rehydration solution regulates radiation‐Induced intestinal barrier disruption in mice. Journal of Nutrition, 150(5), 1100–1108. 32133527 10.1093/jn/nxaa025

[eph70271-bib-0049] Hansen, M. , Bangsbo, J. , Jensen, J. , Krause‐Jensen, M. , Bibby, B. M. , Sollie, O. , Hall, U. A. , & Madsen, K. (2016). Protein intake during training sessions has no effect on performance and recovery during a strenuous training camp for elite cyclists. Journal of the International Society of Sports Nutrition, 13(1), 9. 26949378 10.1186/s12970-016-0120-4 PMC4779585

[eph70271-bib-0050] Hamiel, C. R. , Pinto, S. , & Hau, A. (2009). Glutamine enhances heat shock protein 70 expression via increased hexosamine biosynthetic pathway activity. American Journal of Physiology‐Cell Physiology, 297, 1509–1519. 10.1152/ajpcell.00240.2009 PMC2793053 19776393

[eph70271-bib-0051] Henningsen, K. , Mika, A. , Alcock, R. , Gaskell, S. K. , Parr, A. , Rauch, C. , Russo, I. , Snipe, R. M. J. , & Costa, R. J. (2024). The increase in core body temperature in response to exertional‐heat stress can predict exercise‐induced gastrointestinal syndrome. Temperature, 11(1), 72–91. 10.1080/23328940.2023.2213625 PMC10989703 38577295

[eph70271-bib-0129] Hunter‐Lavin, C. , Davies, E. L. , Bacelar, M. M. , Marshall, M. J. , Andrew, S. M. , & Williams, J. H. (2004). Hsp70 release from peripheral blood mononuclear cells. Biochemical and Biophysical Research Communications, 324(2), 511–517. 15474457 10.1016/j.bbrc.2004.09.075

[eph70271-bib-0052] John, K. , Page, J. , Heffernan, S. M. , Conway, G. E. , Bezodis, N. E. , Kilduff, L. P. , Clark, B. , Périard, J. D. , & Waldron, M. (2024). The effect of a 4‐week, remotely administered, post‐exercise passive leg heating intervention on determinants of endurance performance. European Journal of Applied Physiology, 124(12), 3631–3647. 39052044 10.1007/s00421-024-05558-4 PMC11569002

[eph70271-bib-0053] Kenny, G. P. , & McGinn, R. (2017). Restoration of thermoregulation after exercise. Journal of Applied Physiology, 122(4), 933–944. 27881668 10.1152/japplphysiol.00517.2016

[eph70271-bib-0054] Kephart, W. C. , Wachs, T. D. , Mac Thompson, R. , Brooks Mobley, C. , Fox, C. D. , McDonald, J. R. , Ferguson, B. S. , Young, K. C. , Nie, B. , Martin, J. S. , Company, J. M. , Pascoe, D. D. , Arnold, R. D. , Moon, J. R. , & Roberts, M. D. (2016). Ten weeks of branched‐chain amino acid supplementation improves select performance and immunological variables in trained cyclists. Amino Acids, 48, 779–789. 26553453 10.1007/s00726-015-2125-8

[eph70271-bib-0055] Keirns, B. H. , Koemel, N. A. , Sciarrillo, C. M. , Anderson, K. L. , & Emerson, S. R. (2020). Exercise and intestinal permeability: Another form of exercise‐induced hormesis?. American Journal of Physiology‐Gastrointestinal and Liver Physiology, 319(4), G512–G518. 32845171 10.1152/ajpgi.00232.2020

[eph70271-bib-0056] King, M. A. , Grosche, A. , Ward, S. M. , Ward, J. A. , Sasidharan, A. , Mayer, T. A. , Plamper, M. L. , Xu, X. , Ward, M. D. , Clanton, T. L. , & Vidyasagar, S. (2023). Amino acid solution mitigates hypothermia response and intestinal damage following exertional heat stroke in male mice. Physiological Reports, 11(10), e15681. 37217446 10.14814/phy2.15681 PMC10202825

[eph70271-bib-0057] Kozior, M. , Davies, R. W. , Amigo‐Benavent, M. , Fealy, C. , & Jakeman, P. M. (2023). An Investigation of the protein quality and temporal pattern of peripheral blood aminoacidemia following ingestion of 0.33 g· kg^−1^ body mass protein isolates of whey, pea, and fava bean in healthy, young adult men. Nutrients, 15(19), 4211. 37836496 10.3390/nu15194211 PMC10574361

[eph70271-bib-0058] Krüger, K. , Reichel, T. , & Zeilinger, C. (2019). Role of heat shock proteins 70/90 in exercise physiology and exercise immunology and their diagnostic potential in sports. Journal of Applied Physiology, 126(4), 916–927. 30730806 10.1152/japplphysiol.01052.2018

[eph70271-bib-0059] Labba, I. C. M. , Frøkiær, H. , & Sandberg, A. S. (2021). Nutritional and antinutritional composition of fava bean (*Vicia faba* L., var. minor) cultivars. Food Research International, 140, 110038. 33648264 10.1016/j.foodres.2020.110038

[eph70271-bib-0060] Lee, M. A. , McCauley, R. D. , Kong, S. E. , & Hall, J. C. (2002). Influence of glycine on intestinal ischemia‐reperfusion injury. Journal of Parenteral and Enteral Nutrition, 26(2), 130–135. 11871737 10.1177/0148607102026002130

[eph70271-bib-0061] Luparelli, A. , Trisciuzzi, D. , Schirinzi, W. M. , Caputo, L. , Smiriglia, L. , Quintieri, L. , Nicolotti, O. , & Monaci, L. (2025). Whey proteins and bioactive peptides: Advances in production, selection and bioactivity profiling. Biomedicines, 13(6), 1311. 40564030 10.3390/biomedicines13061311 PMC12189710

[eph70271-bib-0062] Lechner, P. , Buck, D. , Sick, L. , Hemmer, B. , & Multhoff, G. (2018). Serum heat shock protein 70 levels as a biomarker for inflammatory processes in multiple sclerosis. Multiple Sclerosis Journal–Experimental, Translational and Clinical, 4(2), 2055217318767192. 29780609 10.1177/2055217318767192 PMC5954314

[eph70271-bib-0063] Lorenzo, S. , & Minson, C. T. (2007). Human cutaneous reactive hyperaemia: Role of BK_Ca_ channels and sensory nerves. The Journal of physiology, 585(1), 295–303. 17901123 10.1113/jphysiol.2007.143867 PMC2375471

[eph70271-bib-0064] Mack, G. W. (2020). Role of nitric oxide synthase in human sweat gland output. Journal of Applied Physiology, 129(2), 386–391. 32702275 10.1152/japplphysiol.00197.2020

[eph70271-bib-0065] Maharaj, A. , Fischer, S. M. , Dillon, K. N. , Kang, Y. , Martinez, M. A. , & Figueroa, A. (2022). Effects of L‐citrulline supplementation on endothelial function and blood pressure in hypertensive postmenopausal women. Nutrients, 14(20), 4396. 36297080 10.3390/nu14204396 PMC9609406

[eph70271-bib-0066] Morrison, S. A. , Cheung, S. S. , & Cotter, J. D. (2014). Bovine colostrum, training status, and gastrointestinal permeability during exercise in the heat: a placebo‐controlled double‐blind study. Applied Physiology, Nutrition, and Metabolism, 39(9), 1070–1082. 10.1139/apnm-2013-0583 25068884

[eph70271-bib-0067] Multari, S. , Stewart, D. , & Russell, W. R. (2015). Potential of fava bean as future protein supply to partially replace meat intake in the human diet. Comprehensive Reviews in Food Science and Food Safety, 14(5), 511–522.

[eph70271-bib-0068] Mann, G. , Mora, S. , Madu, G. , & Adegoke, O. A. J. (2021). Branched‐chain amino acids: Catabolism in skeletal muscle and implications for muscle and whole‐body metabolism. Frontiers in Physiology, 12, 702826. Frontiers Media S.A. 34354601 10.3389/fphys.2021.702826 PMC8329528

[eph70271-bib-0069] Matomäki, P. , Kainulainen, H. , & Kyröläinen, H. (2018). Corrected whole blood biomarkers–The equation of Dill and Costill revisited. Physiological reports, 6(12), e13749. 29939499 10.14814/phy2.13749 PMC6016620

[eph70271-bib-0070] Mittleman, K. D. , Ricci, M. R. , & Bailey, S. P. (1998). Branched‐chain amino acids prolong exercise during heat stress in men and women. Medicine and science in sports and exercise, 30(1), 83–91. 9475648 10.1097/00005768-199801000-00012

[eph70271-bib-0071] Mee, J. A. , Doust, J. , & Maxwell, N. S. (2015). Repeatability of a running heat tolerance test. Journal of Thermal Biology, 49, 91–97. 25774031 10.1016/j.jtherbio.2015.02.010

[eph70271-bib-0072] Morita, M. , Sakurada, M. , Watanabe, F. , Yamasaki, T. , Ezaki, H. , Morishita, K. , & Miyake, T. (2013). Effects of oral L‐citrulline supplementation on lipoprotein oxidation and endothelial dysfunction in humans with vasospastic angina. Immunology, Endocrine & Metabolic Agents in Medicinal Chemistry, 13(3), 214–220. 10.2174/18715222113139990008 PMC4435567 26005507

[eph70271-bib-0073] Morrison, A. L. , Dinges, M. , Singleton, K. D. , Odoms, K. , Wong, H. R. , & Wischmeyer, P. E. (2006). Glutamine's protection against cellular injury is dependent on heat shock factor‐1. American Journal of Physiology‐Cell Physiology, 290(6), C1625–C1632. 16436470 10.1152/ajpcell.00635.2005

[eph70271-bib-0075] Moura, C. S. , Lollo, P. C. B. , Morato, P. N. , Risso, E. M. , & Amaya‐Farfan, J. (2017). Modulatory effects of arginine, glutamine and branched‐chain amino acids on heat shock proteins, immunity and antioxidant response in exercised rats. Food & Function, 8, 3228–3238. 28812766 10.1039/c7fo00465f

[eph70271-bib-0125] Nielsen, L. L. , Tandrup Lambert, M. N. , & Jeppesen, P. B. (2020). The effect of ingesting carbohydrate and proteins on athletic performance: A systematic review and meta‐analysis of randomized controlled trials. Nutrients, 12(5), Article 1483. 32443678 10.3390/nu12051483 PMC7284704

[eph70271-bib-0076] Newsholme, E. A. , & Blomstrand, E. (2006). Branched‐chain amino acids and central fatigue. The Journal of Nutrition, 136(1), 274S–276S. 16365097 10.1093/jn/136.1.274S

[eph70271-bib-0077] Okazaki, K. , Hayase, H. , Ichinose, T. , Mitono, H. , Doi, T. , & Nose, H. (2009). Protein and carbohydrate supplementation after exercise increases plasma volume and albumin content in older and young men. Journal of Applied Physiology, 107(3), 770–779. 19589953 10.1152/japplphysiol.91264.2008

[eph70271-bib-0078] Oliveira, G. V. D. , Volino‐Souza, M. , Cordeiro, E. M. , Conte‐Junior, C. A. , & Alvares, T. S. (2020). Effects of fish protein hydrolysate ingestion on endothelial function compared to whey protein hydrolysate in humans. International Journal of Food Sciences and Nutrition, 71(2), 242–248. 31271072 10.1080/09637486.2019.1635090

[eph70271-bib-0079] Page, L. K. , Jeffries, O. , & Waldron, M. (2019). Acute taurine supplementation enhances thermoregulation and endurance cycling performance in the heat. European Journal of Sport Science, 19(8), 1101–1109. 30776254 10.1080/17461391.2019.1578417

[eph70271-bib-0080] Page, J. , Scott, G. A. , Aggett, J. N. , Stebbings, G. K. , Kilduff, L. P. , Murphy, C. H. , Waldron, M. , & Heffernan, S. M. (2024). Dietary factors may be associated with measures of ultrasound‐derived skeletal muscle echo intensity. Applied Physiology, Nutrition, and Metabolism, 49(12), 1666–1677. 10.1139/apnm-2024-0256 39178426

[eph70271-bib-0081] Peel, J. , McNarry, A. , Heffernan, S. , Nevola, R. , Kilduff, L. , & Waldron, M. (2025). The effect of dietary supplements on core temperature and sweating responses in hot environmental conditions: A meta‐analysis and meta‐regression. American Journal of Physiology‐Regulatory, Integrative and Comparative Physiology, 328(4), R515–R555. 39884667 10.1152/ajpregu.00186.2024

[eph70271-bib-0082] Peel, J. S. , McNarry, M. A. , Heffernan, S. M. , Nevola, V. R. , Kilduff, L. P. , Coates, K. , Dudley, E. , & Waldron, M. (2024). The effect of 8‐day oral taurine supplementation on thermoregulation during low‐intensity exercise at fixed heat production in hot conditions of incremental humidity. European Journal of Applied Physiology, 124(9), 2561–2576. 38582816 10.1007/s00421-024-05478-3 PMC11365861

[eph70271-bib-0083] Périard, J. D. , Eijsvogels, T. M. , & Daanen, H. A. (2021). Exercise under heat stress: thermoregulation, hydration, performance implications, and mitigation strategies. Physiological reviews, 101(4), 1873–1979. 33829868 10.1152/physrev.00038.2020

[eph70271-bib-0084] Poole, D. C. , Wilkerson, D. P. , & Jones, A. M. (2008). Validity of criteria for establishing maximal O_2_ uptake during ramp exercise tests. European Journal of Applied Physiology, 102(4), 403–410. 17968581 10.1007/s00421-007-0596-3

[eph70271-bib-0085] Pugh, J. N. , Sage, S. , Hutson, M. , Doran, D. A. , Fleming, S. C. , Highton, J. , Morton, J. P. , & Close, G. L. (2017). Glutamine supplementation reduces markers of intestinal permeability during running in the heat in a dose‐dependent manner. European Journal of Applied Physiology, 117, 2569–2577. 29058112 10.1007/s00421-017-3744-4 PMC5694515

[eph70271-bib-0087] Qu, B. , Jia, Y. , Liu, Y. , Wang, H. , Ren, G. , & Wang, H. (2015). The detection and role of heat shock protein 70 in various nondisease conditions and disease conditions: A literature review. Cell Stress and Chaperones, 20(6), 885–892. 26139132 10.1007/s12192-015-0618-8 PMC4595429

[eph70271-bib-0088] Ramanathan, N. L. (1964). A new weighting system for mean surface temperature of the human body. Journal of Applied Physiology, 19(3), 531–533. 14173555 10.1152/jappl.1964.19.3.531

[eph70271-bib-0090] Rao, R. , & Samak, G. (2011). Role of glutamine in protection of intestinal epithelial tight junctions. Journal of Epithelial Biology & Pharmacology, 5(Suppl 1‐M7), 47. 10.2174/1875044301205010047 PMC4369670 25810794

[eph70271-bib-0092] Sawka, M. N. , & Young, A. J. (2006). Physiological systems and their responses to conditions of heat and cold. In C. M. Tipton , M. N. Sawka , C. A. Tate , & R. L. Terjung (Eds.), ACSM's Advanced Exercise Physiology (pp 535–563). Lippincott Williams & Wilkins. Available from: http://www.dtic.mil/dtic/tr/fulltext/u2/a448266.pdf

[eph70271-bib-0093] Schroer, A. B. , Saunders, M. J. , Baur, D. A. , Womack, C. J. , & Luden, N. D. (2014). Cycling time trial performance may be impaired by whey protein and L‐alanine intake during prolonged exercise. International Journal of Sport Nutrition and Exercise Metabolism, 24(5), 507–515. 24937205 10.1123/ijsnem.2013-0173

[eph70271-bib-0095] Sepponen, K. , & Pösö, A. R. (2006). The inducible form of heat shock protein 70 in the serum, colon and small intestine of the pig: comparison to conventional stress markers. Veterinary Journal, 171(3), 519–524. 16624719 10.1016/j.tvjl.2005.01.005

[eph70271-bib-0096] Singleton, K. D. , & Wischmeyer, P. E. (2006). Oral glutamine enhances heat shock protein expression and improves survival following hyperthermia. Shock, 25(3), 295–299. 16552363 10.1097/01.shk.0000196548.10634.02

[eph70271-bib-0097] Simmons, G. H. , Wong, B. J. , Holowatz, L. A. , & Kenney, W. L. (2011). Changes in the control of skin blood flow with exercise training: where do cutaneous vascular adaptations fit in? Experimental Physiology, 96(9), 822–828. 21602295 10.1113/expphysiol.2010.056176 PMC3754812

[eph70271-bib-0098] Snipe, R. M. , Khoo, A. , Kitic, C. M. , Gibson, P. R. , & Costa, R. J. (2017). Carbohydrate and protein intake during exertional heat stress ameliorates intestinal epithelial injury and small intestine permeability. Applied Physiology, Nutrition, and Metabolism, 42(12), 1283–1292. 10.1139/apnm-2017-0361 28777927

[eph70271-bib-0099] Tataka, Y. , Haramura, M. , Hamada, Y. , Ono, M. , Toyoda, S. , Yamada, T. , Hiratsu, A. , Suzuki, K. , & Miyashita, M. (2022). Effects of oral cystine and glutamine on exercise‐induced changes in gastrointestinal permeability and damage markers in young men. European Journal of Nutrition, 61(5), 2331–2339. 35106632 10.1007/s00394-022-02806-1 PMC9279189

[eph70271-bib-0101] Thijssen, D. H. , Bruno, R. M. , van Mil, A. C. , Holder, S. M. , Faita, F. , Greyling, A. , Zock, P. , Taddeo, S. , Deanfield, J. , Luscher, T. , Green, D. , & Ghiadoni, L. (2019). Expert consensus and evidence‐based recommendations for the assessment of flow‐mediated dilation in humans. European Heart Journal, 40(30), 2534–2547. 31211361 10.1093/eurheartj/ehz350

[eph70271-bib-0102] Tumilty, L. , Davison, G. , Beckmann, M. , & Thatcher, R. (2011). Oral tyrosine supplementation improves exercise capacity in the heat. European Journal of Applied Physiology, 111(12), 2941–2950. 21437603 10.1007/s00421-011-1921-4

[eph70271-bib-0103] Tyler, C. J. , Coffey, T. R. M. , & Hodges, G. J. (2016). Acute l‐arginine supplementation has no effect on cardiovascular or thermoregulatory responses to rest, exercise, and recovery in the heat. European Journal of Applied Physiology, 116(2), 363–371. 26563117 10.1007/s00421-015-3295-5

[eph70271-bib-0104] Van Wijck, K. , Wijnands, K. A. , Meesters, D. M. , Boonen, B. , Van Loon, L. J. , Buurman, W. A. , Dejong, C. H. , Lenaerts, K. , & Poeze, M. (2014). L‐citrulline improves splanchnic perfusion and reduces gut injury during exercise. Medicine & Science in Sports & Exercise, 46(11), 2039–2046. 24621960 10.1249/MSS.0000000000000332

[eph70271-bib-0105] Varasteh, S. , Braber, S. , Kraneveld, A. D. , Garssen, J. , & Fink‐Gremmels, J. (2018). l‐Arginine supplementation prevents intestinal epithelial barrier breakdown under heat stress conditions by promoting nitric oxide synthesis. Nutrition Research, 57, 45–55. 30122195 10.1016/j.nutres.2018.05.007

[eph70271-bib-0106] Wang, B. , Wu, G. , Zhou, Z. , Dai, Z. , Sun, Y. , Ji, Y. , Li, W. , Wang, W. , Liu, C. , Han, F. , & Wu, Z. (2015). Glutamine and intestinal barrier function. Amino Acids, 47, 2143–2154. 24965526 10.1007/s00726-014-1773-4

[eph70271-bib-0107] Wang, Y. , Lin, F. , Zhu, X. , Leone, V. A. , Dalal, S. , Tao, Y. , Messer, J. S. , & Chang, E. B. (2018). Distinct roles of intracellular heat shock protein 70 in maintaining gastrointestinal homeostasis. American Journal of Physiology‐Gastrointestinal and Liver Physiology, 314(2), G164–G178. 29051186 10.1152/ajpgi.00208.2017 PMC5866418

[eph70271-bib-0108] Wang, W. W. , Qiao, S. Y. , & Li, D. F. (2009). Amino acids and gut function. Amino Acids, 37, 105–110. 18670730 10.1007/s00726-008-0152-4

[eph70271-bib-0109] Watson, P. , Shirreffs, S. M. , & Maughan, R. J. (2004). The effect of acute branched‐chain amino acid supplementation on prolonged exercise capacity in a warm environment. European Journal of Applied Physiology, 93(3), 306–314. 15349784 10.1007/s00421-004-1206-2

[eph70271-bib-0110] Williams, M. (2005). Dietary supplements and sports performance: Amino acids. Journal of the International Society of Sports Nutrition, 2(2), 63. 18500957 10.1186/1550-2783-2-2-63 PMC2129148

[eph70271-bib-0111] Wu, G. , Meininger, C. J. , McNeal, C. J. , Bazer, F. W. , & Rhoads, J. M. (2021). Role of L‐arginine in nitric oxide synthesis and health in humans. In Amino acids in nutrition and health (pp. 167–187). Springer. 10.1007/978-3-030-74180-8_10 34251644

[eph70271-bib-0112] Yamada, P. M. , Amorim, F. T. , Moseley, P. , Robergs, R. , & Schneider, S. M. (2007). Effect of heat acclimation on heat shock protein 72 and interleukin‐10 in humans. Journal of Applied Physiology, 103(4), 1196–1204. 17615280 10.1152/japplphysiol.00242.2007

[eph70271-bib-0113] Yin, L. , Gupta, R. , Vaught, L. , Grosche, A. , Okunieff, P. , & Vidyasagar, S. (2016). An amino acid‐based oral rehydration solution (AA‐ORS) enhanced intestinal epithelial proliferation in mice exposed to radiation. Scientific Reports, 6, 37220. 27876791 10.1038/srep37220 PMC5120277

[eph70271-bib-0114] Young, P. , Russo, I. , Gill, P. , Muir, J. , Henry, R. , Davidson, Z. , & Costa, R. J. (2023). Reliability of pathophysiological markers reflective of exercise‐induced gastrointestinal syndrome (EIGS) in response to 2‐h high‐intensity interval exercise: A comprehensive methodological efficacy exploration. Frontiers in Physiology, 14, 1063335. 36895638 10.3389/fphys.2023.1063335 PMC9989174

[eph70271-bib-0116] Zhang, H. , Huizenga, C. , Arens, E. , & Wang, D. (2004). Thermal sensation and comfort in transient non‐uniform thermal environments. European Journal of Applied Physiology, 92, 728–733. 15221406 10.1007/s00421-004-1137-y

[eph70271-bib-0117] Zhang, J. , Bhattacharyya, S. , Hickner, R. C. , Light, A. R. , Lambert, C. J. , Gale, B. K. , Fiehn, O. , & Adams, S. H. (2019). Skeletal muscle interstitial fluid metabolomics at rest and associated with an exercise bout: Application in rats and humans. American Journal of Physiology‐Endocrinology and Metabolism, 316(1), E43–E53. 30398905 10.1152/ajpendo.00156.2018 PMC6417688

[eph70271-bib-0126] Zhao, S. , Zhang, H. , Xu, Y. , Li, J. , Du, S. , & Ning, Z. (2024). The effect of protein intake on athletic performance: a systematic review and meta‐analysis. Frontiers in Nutrition, 11, 1455728. 39628467 10.3389/fnut.2024.1455728 PMC11613885

[eph70271-bib-0118] Zuhl, M. N. , Lanphere, K. R. , Kravitz, L. , Mermier, C. M. , Schneider, S. , Dokladny, K. , & Moseley, P. L. (2014). Effects of oral glutamine supplementation on exercise‐induced gastrointestinal permeability and tight junction protein expression. Journal of Applied Physiology, 116(2), 183–191. 24285149 10.1152/japplphysiol.00646.2013 PMC3921361

[eph70271-bib-0119] Zuhl, M. , Dokladny, K. , Mermier, C. , Schneider, S. , Salgado, R. , & Moseley, P. (2015). The effects of acute oral glutamine supplementation on exercise‐induced gastrointestinal permeability and heat shock protein expression in peripheral blood mononuclear cells. Cell Stress and Chaperones, 20, 85–93. 25062931 10.1007/s12192-014-0528-1 PMC4255255

