# Supplementary material for: Plant‐protein supplementation improves thermoregulatory responses and ameliorates markers of intestinal damage during exercise in the heat
Source: Exp Physiol. 2026 Mar 31:10.1113/EP093504. Online ahead of print. doi: 10.1113/EP093504 (PMC13394769; doi:10.1113/EP093504)
Supplement: Supplementary file 1 — Supporting Information [file EPH-9999-0-s001.docx]

**Supplementary material**

**Supplementary file 1**

**Table 1.** Results from the 3-day weighed food diary during each supplementation period.

| **Components** | **Placebo** | **Protein** | **p-value** |
| --- | --- | --- | --- |
| Energy (kcal) | 1969.95 $\pm$ 651 | 1690.89 $\pm$ 585 | 0.121 |
| Carbs (g) | 215.32 $\pm$ 69.3 | 195.00 $\pm$ 70.8 | 0.131 |
| Protein (g) | 96.13 $\pm$ 46.5 | 95.86 $\pm$ 48.3 | 0.118 |
| Fat (g) | 80.35 $\pm$ 49.7 | 58.57 $\pm$ 26.1 | 0.973 |
| **Amino Acids (mg)** |  |  |  |
| Tryptophan | 838.57 $\pm$ 493.68 | 810.10 $\pm$ 477.36 | 0.801 |
| Isoleucine | 2228.38 $\pm$ 1709.97 | 2275.45 $\pm$1784.41 | 0.464 |
| Leucine | 3657.42 $\pm$2800.57 | 3798.65 $\pm$3046.86 | 0.932 |
| Lysine | 3018.25 $\pm$2800.62 | 3295.24 $\pm$3353.57 | 0.944 |
| Methionine | 1091.06 $\pm$926.81 | 1174.85 $\pm$996.21 | 0.956 |
| Cysteine | 685.78 $\pm$482.92 | 679.97 $\pm$476.01 | 0.906 |
| Phenylalanine | 2167.26 $\pm$1626.81 | 2167.72 $\pm$1551.78 | 0.878 |
| Glutamic Acid | 8215.44 $\pm$5820.92 | 8393.33 $\pm$5960.95 | 0.769 |
| Tyrosine | 1637.29 $\pm$1292.44 | 1665.49 $\pm$1333.40 | 0.895 |
| Threonine | 1918.98 $\pm$1521.86 | 2008.59 $\pm$1679.16 | 0.822 |
| Valine | 2649.16 $\pm$2002.79 | 2732.27 $\pm$1969.35 | 0.657 |
| Histidine | 1297.71 $\pm$1105.93 | 1372.35 $\pm$1290.48 | 0.655 |
| Arginine | 2798.45 $\pm$2408.26 | 2845.51 $\pm$2551.68 | 0.999 |
| Aspartic Acid | 4278.95 $\pm$3442.73 | 4329.24 $\pm$3736.14 | 0.943 |
| Proline | 2767.41 $\pm$2014.28 | 2732.28 $\pm$1572.63 | 0.880 |
| Serine | 2506.54 $\pm$1828.17 | 2447.22 $\pm$1660.59 | 0.782 |
| Glycine | 1832.51 $\pm$1652.23 | 1894.06 $\pm$1812.63 | 0.919 |
| Alanine | 2495.90 $\pm$2386.45 | 2364.73 $\pm$2227.48 | 0.841 |

**Supplementary file 2**

**Table 2.** Mean differences (95% confidence interval; CI) (ATURA − Placebo).

|  | **Outcome** | **Mean difference** | **95% CI** | **Units** |
| --- | --- | --- | --- | --- |
| Exercise responses | |  |  |  |
|  | GXT duration | 0.33 | -1.03, 1.69 | min |
|  | Thermo $\dot{V}$O_2peak_ | -1.20 | -6.86, 4.57 | ml/kg/min |
|  | $\dot{V}$O_2peak_ | -0.80 | -5.52, 3.78 | ml/kg/min |
| *Cardiometabolic responses* | |  |  |  |
|  | Heart rate | -0.59 | -3.47, 2.28 | beats/min |
|  | Oxygen consumption | 80.73 | -125.23, 286.68 | mL/min |
|  | Respiratory exchange ratio | -0.03 | −0.03, 0.03 | - |
|  | Plasma volume | -1.49 | -3.98, 1.01 | % |
| *Thermoregulatory responses* | |  |  |  |
|  | Whole body sweat rate | 2.49 | 0.20, 3.38 | g/min |
|  | Local sweat rate | 110 | -41.65, 286.71 | nL/min |
|  | Core temperature | -0.14 | -0.46, 0.19 | °C |
|  | Skin temperature | 0.30 | -0.59, 0.79 | °C |
| *Perceptual responses* | |  |  |  |
|  | Rating of perceived exertion | -0.08 | -0.51, 0.35 | AU |
|  | Thermal sensation | -0.33 | -0.45, -0.02 | AU |
|  | Thermal comfort | -0.04 | -0.24, 0.12 | AU |
| *Partitional calorimetry and skin blood flow* | |  |  |  |
|  | Calculated skin blood flow | 0.080 | -0.676, 0.52 | L/min |
|  | Heat balance | 17.50 | -157.50 to 122.61 | Watts |
|  | evaporation at the skin’s surface | 130.50 | -396.0, 135.0 | Watts |
|  | Heat production | 18.30 | -148.0, 111.4 | Watts |
|  | Heat storage | -153.40 | -75.71, 382.5 | Watts |
|  | Dry heat loss | -0.14 | -9.26, 9.54 | Watts |
| Vascular responses | |  |  |  |
|  | Flow mediated dilation | 0.05 | -0.74, 0.84 | % |
|  | Baseline diameter | -0.02 | -0.08, 0.04 | mm |
|  | Peak diameter | 0.04 | -0.08, 0.17 | mm |
|  | Baseline shear rate | 4.80 | -16.60, 26.22 | s^-1^, AUC |
|  | Peak shear rate | 5.0 | -106.60, 116.60 | s^-1^, AUC |
| *Gastrointestinal responses* | |  |  |  |
|  | Total GIS | -16 | -43, 11 | % |
|  | Upper GIS | -4 | -30, 22 | % |
|  | Lower GIS | -12 | -3, 17 | % |
|  | Other | 1 | -10, 12 | % |
| *Circulating biomarkers* (post-exercise) | |  |  |  |
|  | HSP70 | 89.42 | -29.97, 208.80 | pg/mL |
|  | IL-6 | -38.45 | -182.10, 105.21 | pg/mL |
|  | i-FABP | -189.90 | -389.5, 9.70 | pg/mL |
|  | sCD14 | -8.18 | -19.28, 2.91 | ng/mL |

*GXT; graded exercise test, GIS; gastrointestinal symptoms, V̇O₂peak; peak oxygen consumption, HSP70; heat shock protein 70, IL6; interleukin-6, i-FABP; intestinal fatty acid-binding protein, sCD14; soluble cluster of differentiation 14.*
